# Chimeric antigen receptor T (CAR-T) cell therapy in non-oncological diseases

**DOI:** 10.1038/s41392-026-02783-5

**Published:** 2026-10-01

**Authors:** Zhuolin Fan, Xu Zheng, Chuan Jin, Jingting Zhang, Ningqiang Gong, Xuexin Li

**Affiliations:** 1 Department of General Surgery, The Fourth Affiliated Hospital of China Medical University, Shenyang, China; 2 Biomedical Innovation Center, The Fourth Affiliated Hospital of China Medical University, Shenyang, China; 3 Department of Immune, Gene and Pathology, Uppsala University, Uppsala, Sweden; 4 College of Management, Liaoning Economy Vocational and Technical College, Shenyang, China; 5 Department of Nutrition and Food Hygiene, School of Pubilc Health, China Medical University, Shenyang, China; 6 Division of Life Sciences and Medicine, University of Science and Technology of China, Hefei, China; 7 Department of Physiology and Pharmacology, Karolinska Institute, Solna, Sweden

**Keywords:** Biotechnology, Drug development

## Abstract

The application of chimeric antigen receptor T (CAR-T) cells in hematologic malignancies has driven significant advancements in this form of immunotherapy. The therapeutic strategy of CAR-T cells targeting specific cell populations has opened new avenues for treating non-oncological diseases, such as autoimmune diseases, aging-related conditions, and infections. For instance, in non-oncological diseases like systemic lupus erythematosus (SLE), abnormal B cell development or dysfunction leads to the production of autoantibodies, triggering localized deposition of immune complexes and resulting in tissue or organ damage and dysfunction. Relevant studies have identified disease-specific surface antigens or pathogenic cell subsets, making CAR-T cell therapy a feasible treatment approach. Moreover, in non-oncological diseases, CAR-T cells can mediate immune remodeling for certain conditions, thereby achieving long-term therapeutic remission. Although no CAR-T therapies have yet been approved for non-oncological diseases, multiple clinical trials have been initiated, with some achieving interim successes (e.g., allogeneic CAR-T cells have demonstrated efficacy in treating rheumatic diseases). Meanwhile, ongoing research into the pathogenesis of non-neoplastic diseases further supports the potential application of CAR-T cells. With this background, this article aims to introduce the latest research, mechanisms, and applications of CAR-T cell therapy in non-oncological diseases, summarize the pathogenesis of related disorders, and discuss the advantages, challenges, and future prospects of CAR-T cell therapy.

## Introduction

Chimeric Antigen Receptor T (CAR-T) cells are genetically engineered T lymphocytes expressing synthetic transmembrane receptors that confer antigen-specific targeting capabilities. Structurally, these chimeric receptors integrate an extracellular antigen-binding domain (typically a single-chain fragment variable, scFv) with intracellular signaling modules capable of activating T cell effector functions. Upon antigen engagement, CAR-T cells initiate cytotoxic programs through perforin-granzyme secretion and Fas/FasL pathways, while concurrently recruiting innate immune components via cytokine release (e.g., interferon (IFN)-γ, interleukin (IL)-2), thereby orchestrating coordinated antitumor immunity.^1–5^ The conceptual foundation of CAR technology emerged from early attempts to reconfigure immune receptor architectures. In 1987, TCR-antibody fusion enabled MHC-independent antigen recognition, decoupling specificity from TCR signaling.^6,7^ By 1989, chimeric TCRs targeting folate-binding protein lysed tumor cells, validating engineered T-cell therapy.^8,9^ In 1993, Eshhar et al. created the first CAR by linking an anti-TNP scFv to CD3ζ, combining antibody-like targeting with TCR activation. This modular design paved the way for later CAR generations with costimulatory domains (e.g., CD28, 4-1BB).^10–12^

The clinical application of chimeric antigen receptor (CAR) T-cell therapy achieved a major milestone in 2017 with the first Food and Drug Administration (FDA) approval of tisagenlecleucel for B-cell malignancies. As of November 2024, regulatory agencies including both the U.S. FDA and European Medicines Agency (EMA) have approved seven distinct CAR-T cell products. These therapies primarily target two key antigens: CD19 for B-cell malignancies (including relapsed/refractory diffuse large B-cell lymphoma (DLBCL), follicular lymphoma, mantle cell lymphoma (MCL), and B-cell acute lymphoblastic leukemia (ALL)), and B-cell maturation antigen (BCMA) for relapsed/refractory multiple myeloma.^13–24^ Mechanistically, CAR-T cells recognize CD19 or BCMA via their engineered chimeric receptors, initiating dual cytotoxic pathways: (1) perforin/granzyme-mediated direct target cell lysis, and (2) Fas/FasL interaction-induced apoptosis. Concurrently, activated CAR-T cells secrete pro-inflammatory cytokines (e.g., IFN-γ, Tumor necrosis factor (TNF)-α), recruiting innate immune effectors (macrophages, Natural killer (NK) cells) to amplify tumoricidal activity. This multi-modal attack achieves sustained depletion of malignant CD19^+^ B-cell populations.^25^

The transformative success of CAR-T cell therapy in oncology has catalyzed its exploration across immune-mediated pathologies, leveraging its precision targeting to reprogram aberrant immune activity. In autoimmune conditions, pathogenic B cells play a central role by producing autoantibodies that form immune complexes, thereby driving aberrant immune activation and tissue damage.^26^ Beyond autoimmunity, CAR-T cells may also address other pathological processes: senescent cells, which exacerbate inflammation through senescence-associated secretory phenotypes (SASP)^27^; cardiomyocyte injury, which triggers fibroblast activation and progressive fibrosis^28^; and chronic infections (e.g., HIV, HBV, Aspergillus), which perpetuate immune dysregulation. In the context of infectious diseases like HIV and HCMV, where immune deficiency dominates the clinical picture, CAR-T cells are designed to directly eliminate infected cells and restore immune competence.^29,30^ Similarly, in graft-versus-host disease (GvHD)—a complication of allogeneic transplantation—regulatory CAR-T cells hold promise for suppressing excessive alloimmune responses while preserving protective immunity.^31^ This expanding repertoire of applications underscores the versatility of CAR-T technology in recalibrating disrupted immune homeostasis across diverse disease states.^32–34^

While CAR-T therapy offers distinct advantages for non-malignant diseases, including potential long-term remission through immune memory formation^35^ and reduced cytokine release syndrome risk compared to cancer applications, several challenges remain: Target specificity: Many senescence-associated antigens are also expressed on healthy tissues.^36^ Precision requirements: Chronic diseases demand exceptionally specific targeting to avoid off-tissue effects. Cost barriers: High expenses associated with autologous/allogeneic manufacturing.^37^

This article comprehensively examines CAR-T applications across non-oncologic diseases, including: autoimmune disorders, infectious diseases, aging and senescence, cardiovascular and metabolic disorders and neurological diseases. Analyzing their design principles, mechanisms of action, therapeutic potential, the aim is to provide insights into target selection, pathogenesis research, CAR design optimization, and strategies for enhancing the safety and controllability of CAR-T cell therapy in non-oncological diseases. This review seeks to contribute to the clinical application and broader adoption of CAR-T cells in non-oncological diseases.

## Structure and mechanism of CAR-T

### Structure and generations of CARs

**Fig. 1:**
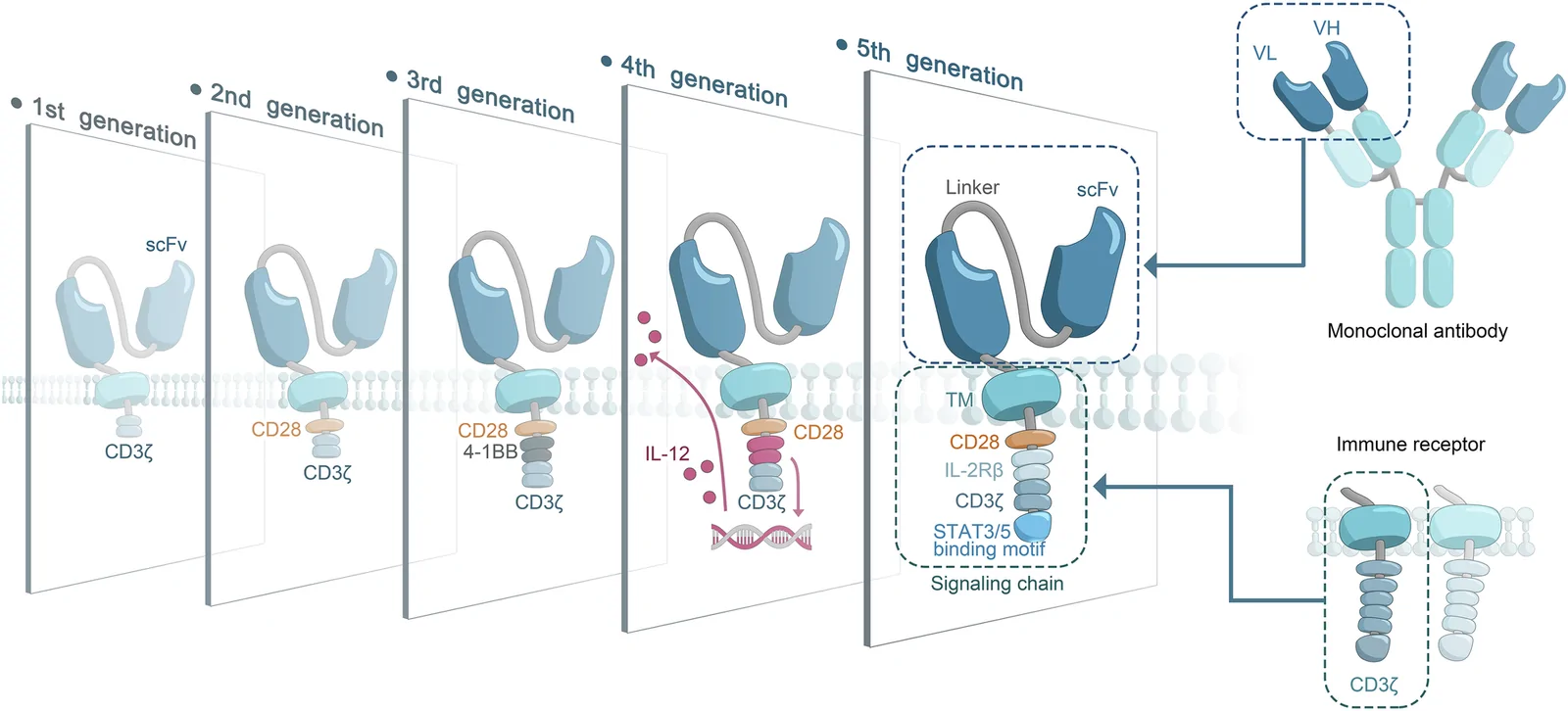
CAR structure and iteration diagram. scFv: single-chain variable fragment, the smallest functional fragment with full antigen activity, containing the antibody recognition and antigen-binding sites; CD3ζ: cluster of differentiation 3, the CD3ζ chain typically exists as a ζ-ζ homodimer connected to the intracellular segment of the T cell receptor (TCR) and associates non-covalently with the TCR to form the TCR/CD3 receptor complex. In this complex, CD3 is responsible for mediating the transmission of signals from the extracellular domain to the intracellular domain upon TCR stimulation, thereby activating intracellular signal transduction; TM: transmembrane domain. 1st generation CAR-T cells use intracellular CD3ζ as the signaling domain, with an extracellular scFv domain that recognizes and binds to the target antigen. 2nd generation CAR-T cells incorporate an additional co-stimulatory domain, such as CD28, in the intracellular structure, enhancing the activation and persistence of the engineered T cells. 3rd generation CAR-T cells build upon the 2nd generation by adding another co-stimulatory domain, such as 4-1BB, further synergistically enhancing T cell activation and persistence. 4th generation, also named as TRUCKs, are genetically engineered to secrete cytokines, such as IL-12, which recruit NK cells and macrophages to kill target cells that are not directly bound by the CAR. This generation also incorporates various gene regulatory elements through genetic modification to further enhance the function of CAR-T cells. 5th generation CAR-T cells introduce intracellular co-stimulatory structures that activate the JAK-STAT signaling pathway, thereby enhancing the cytotoxic function of CAR-T cells. TRUCKs T cells Redirected for Antigen-Unrestricted Cytokine-mediated Killing, NK cells natural killing cells

Chimeric antigen receptors (CARs) are fusion proteins consisting of an intracellular signal transduction domain (such as CD3ζ), a transmembrane domain, and an extracellular domain which is single-chain variable fragment (scFv) derived from the antibody. The specificity of CARs is mainly determined by the extracellular scFv domain, which specifically recognizes target cells, followed by transmitting activation signal to CAR-T cells for cytotoxic effects.^11,12,38^ (Fig. 1).

The development of CAR constructs has evolved through five distinct generations, each addressing critical limitations of previous designs. First-generation CARs, incorporating only the CD3ζ signaling domain, demonstrated restricted clinical efficacy primarily due to insufficient T cell expansion and rapid exhaustion upon repeated antigen exposure.^39^ This key limitation prompted the development of second-generation CARs, which incorporated additional co-stimulatory domains (CD28, 4-1BB, or OX40) alongside CD3ζ, resulting in significantly enhanced T cell activation, expansion, cytotoxicity, and persistence.^40–45^ Building on this advancement, third-generation CARs combined multiple co-stimulatory domains (typically CD3ζ, CD28, and 4-1BB) to further optimize signaling pathways, though comparative studies have shown variable impacts on in vivo persistence depending on domain combinations.^46,47^ The emergence of fourth-generation CARs, termed TRUCKs (T cells Redirected for Antigen-Unrestricted Cytokine-mediated Killing), introduced two major innovations: (1) capacity for cytokine secretion (particularly IL-12) to recruit and activate innate immune cells against antigen-negative tumor cells, and (2) incorporation of safety mechanisms including suicide genes and logic-gated control systems for enhanced regulation. Most recently, fifth-generation CARs have integrated novel co-stimulatory domains capable of activating JAK-STAT signaling pathways, offering potential advantages in T cell proliferation and long-term persistence.^38,46,48–54^ This progressive evolution of CAR designs reflects an ongoing effort to balance potent antitumor activity with improved safety and durability of cellular responses (Fig. 1).

### Engineering strategies

This therapeutic gap has driven intensive research into optimizing the chimeric antigen receptor (CAR) structure itself, with several innovative strategies currently under investigation to enhance precision and functionality. A key advancement involves the development of logic-gated systems, where CAR activation requires specific input combinations (OR, AND, or NOT gates).^55–60^ These synthetic biology approaches create multi-signal responsive circuits that improve targeting accuracy while maintaining system reliability—if one activation pathway fails, alternative pathways can still trigger the desired response.^55–60^ Such OR logic gates can be implemented through various designs including bivalent,^55–57^ bicistronic,^58^ two vectors,^59^ co-infusion.^60^

Parallel efforts focus on cellular fitness enhancement through genetic modifications. These include overexpression of pro-survival factors like c-Jun,^61^ knockout of inhibitory receptors (PD1),^62^ TGFBR2,^63^ HPK1 (also known as MAP4K1)^64^), and incorporation of safety switches (EGFRt,^65^ CD20 epitope,^66^ HER2t,^67^ HSV-tk,^68^ iCasp9^69^) for controlled elimination if needed. Regulatable platforms represent another critical innovation, enabling precise spatiotemporal control of CAR activity through external inducers like AP1903-inducible costimulation,^70^ antibody-coupled,^71^ fluorescein-CAR,^72^ switchable CAR T cells,^73^ or antigen receptor complex (ARC) T cells.^74^

To combat the immunosuppressive tumor microenvironment, “armored” CAR-T cells have been engineered with enhanced defensive capabilities. These include dominant-negative receptors (TGFβR,^75^ PD-1,^76^ FAS^77^), checkpoint inhibitors (PD-1-Fc, anti-PD-1 scFv^78,79^), cytokine modifications (IL-12,^80^ IL-18,^81^ NFAT-induced IL-12,^82^ IL-15 and IL-21^83^), and switch receptors (PD-1–CD28, IL-4R–IL-2Rβ^84^). Complementary strategies aim to engage endogenous immunity through chemokine receptor overexpression (CXCR5, CCR4, CCL19, CCR2, CX3CR1^85,86^) or immune modulator expression (CD40L overexpression^87^).

Finally, improving CAR-T cell expansion and persistence remains a major focus, with approaches including IL-15 secretion,^88^ sushi domain,^89^ tethered,^90^ IL-7: secretion,^91^ or mutant constitutive.^92^ These multifaceted optimization strategies aim to overcome current limitations and expand CAR-T therapy’s potential across broader disease indications.

## Mechanisms of action in non-oncological diseases

### Mechanism of CAR-T cells

**Fig. 2:**
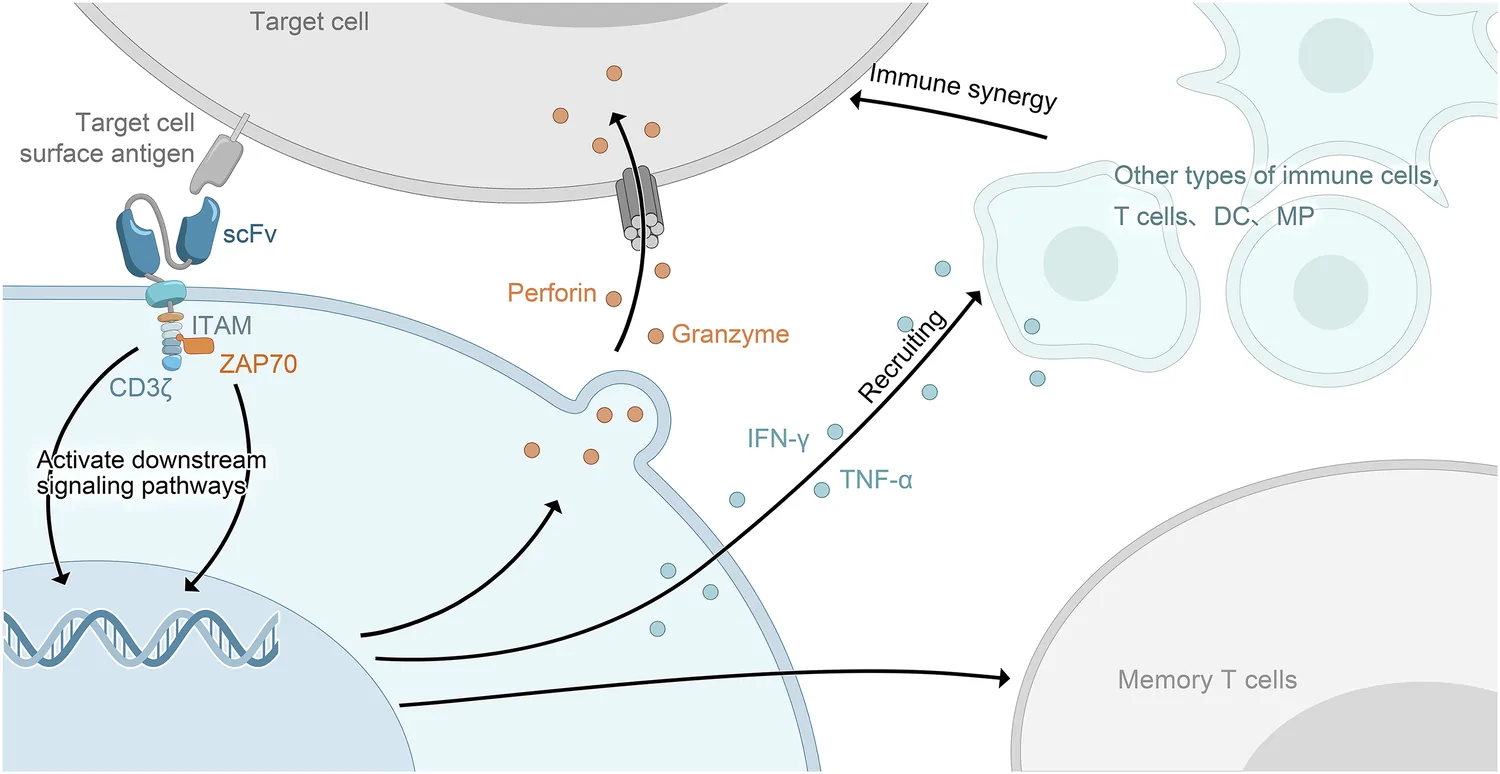
Mechanism of CAR-T to target cells. After the extracellular scFv domain of the CAR binds to the antigen on the surface of the target cell, the intracellular CD3ζ domain mediates ITAM phosphorylation. This is followed by signal transduction via ZAP70 to the intracellular signaling domains, leading to CAR-T cell activation and the initiation of immune responses. These immune effects occur primarily through: direct secretion of perforin and granzymes, which induce target cell death; production of cytokines that recruit and activate other immune cells such as macrophages, T cells, and dendritic cells at the target site, triggering a localized immune response; generation of memory T cells, which enable a rapid immune response upon re-exposure to the target antigen. ITAM: immunoreceptor tyrosine-based activation motif; ZAP70: 70 kDa c-related protein

CAR-T cells exert therapeutic effects through an antigen-driven activation cascade (Fig. 2). Target recognition begins when the CAR’s scFv domain engages cognate antigens, triggering intracellular signaling via costimulatory domains (CD28/4-1BB) and CD3ζ ITAMs.^40,42,43,93–95^ This initiates three core effector mechanisms^96,97^: 1. direct Cytotoxicity: perforin/granzyme-mediated pore formation and caspase activation; and Fas-FasL induced apoptosis^98^; 2. immune modulation includes: secretion of IFN-γ and TNF-α promote the infiltration of monocytes/macrophages while enhancing macrophage activation and the secretion of chemokines such as CCL2. These cytokines also stimulate IL-12 production, which activates downstream signaling pathways to induce IFN-γ secretion by T cells and NK cells. The released IFN-γ further amplifies the activation of macrophages and dendritic cells (DCs), establishing a positive feedback loop that sustains and intensifies the immune response,^99,100^ and establishment of pro-inflammatory microenvironments; 3. immunological memory formation: generation of central/effector memory T cell subsets for sustained surveillance.^101^

The functional heterogeneity among T cell subsets (memory T cells and effector T cells) within individual patients provides critical insights for the efficacy of CAR-T cell immunotherapy. T cells mature in the thymus and express T cell receptors (TCRs). When expressing CD8 on their surface, they are termed CD8^+^ T cells (cytotoxic T cells), while those expressing CD4 are called CD4^+^ T cells (helper T cells).^102^ CD4^+^ T cells further differentiate into distinct subsets: Th1, Th2, Th9, Th17, Th22, Treg (regulatory T cells), and Tfh (follicular helper T cells), each characterized by unique cytokine profiles that play pivotal roles in immune and effector responses. All CD4^+^ Th subsets originate from naive CD4^+^ T cells through specific cytokine induction: Th1 differentiation is driven by IL-12 and IFN-γ, Th2 by IL-4, and Treg by IL-2 and TGF-β.^103,104^ Each Th subset releases characteristic cytokines that mediate pro-inflammatory, anti-inflammatory, survival, or protective functions. For instance, Th1 cells secrete IFN-γ and TNF; Th2 cells produce IL-4 (a crucial survival factor for B lymphocytes), IL-5 and IL-13; Th9 generates IL-9; Treg secretes IL-10 (an immunosuppressive cytokine that maintains FOXP3 transcription factor expression required for Treg-mediated suppression) and TGF-β; while Th17 produces IL-17 (a cytokine essential for host defense against bacteria and fungi).^105^

Notably, cytokines from Th1 and Th2 CD4^+^ T cell subsets have been shown to differentially modulate cytotoxicity in second-generation CD28-costimulated CAR-T cells—high concentrations of Th1 cytokines induce acute toxicity, whereas elevated Th2-type cytokines cause chronic autotoxicity in animals receiving CD19-targeted CAR-T therapy. Another example involves engineered CAR-T cells producing inducible IL-12, which recruits macrophages, reprograms immunosuppressive cells, and modifies tumor stroma to enhance tumor killing.^106^

T cell differentiation, particularly the balance between memory and effector T cells, plays a fundamental role in pathogen-directed immunity. Effector cells are short-lived, while memory cells possess long-term survival potential. The persistence of CAR-T therapy has been demonstrated to depend on the infusion product’s content of CD4^+^ cells and central memory cells (CD45RO^+^CD62L^+^).^107^ Naive CD8^+^ T cells can differentiate into stem cell memory T cells (TSCM), central memory T cells (TCM), effector memory T cells (TEM), and terminal effector cells (TEFF). During CD8^+^ T cell differentiation, effector functions increase while memory potential and proliferative capacity decline, with distinct biomarkers including L-selectin, CD45RO, CD45RA, and CCR-7 marking different stages.^108^ Non-human primate studies have shown that CD8^+^ clones derived from central memory T cells (rather than effector cells) exhibit prolonged persistence after adoptive transfer.^109^ Furthermore, combined CD8^+^ and CD4^+^ subsets significantly enhance T cell transfer efficacy, with CD4^+^ cells supporting the development of CD8^+^ memory function. These findings underscore the importance of specific T cell subsets and their combinations in immunotherapy.

### Immune reconstitution

CAR-T cell therapy represents a transformative approach for immune reconstitution in autoimmune diseases through two complementary mechanisms: targeted depletion of autoreactive lymphocytes and restoration of regulatory functions. The pathogenesis of autoimmune disorders stems from complex interactions between genetic predisposition and environmental triggers that disrupt immune tolerance.^110–113^ This breakdown manifests most prominently as aberrant activation of B and T lymphocytes against self-antigens, resulting in pathogenic autoantibody production. These autoantibodies form immune complexes with self-antigens, triggering inflammatory cascades that ultimately cause tissue damage. The precision of CAR-T technology enables selective targeting of these autoreactive lymphocytes through chimeric antigen receptors designed to recognize lymphocyte surface markers. Upon engagement, CAR-T cells induce apoptosis of pathogenic B and T cells through both direct cytotoxicity (perforin/granzyme-mediated lysis) and death receptor signaling. Clinical applications demonstrate this strategy’s potential: CD19-directed CAR-T cells have achieved durable B-cell depletion and clinical improvement in systemic lupus erythematosus (SLE),^114,115^ and KYV-101, a CD19-targeting CAR-T product, has shown efficacy in depleting pathogenic lymphocytes in both multiple sclerosis and rheumatoid arthritis.^116–118^

Equally important is CAR-T therapy’s capacity to augment impaired regulatory mechanisms. The immune system maintains homeostasis through balanced activation and suppression, and autoimmune diseases often feature regulatory T cell (Treg) dysfunction. Engineered CAR-Tregs address this defect by: suppressing pathogenic cytokine production (e.g., IL-23R CAR-Tregs reduce IFN-γ and TNF in inflammatory bowel disease).^119,120^ Controlling alloreactive responses in graft-versus-host disease through HLA-A2-specific, CD19-targeted, or OX40L-directed CAR-Tregs.^121–124^ These regulatory CAR-T cells promote long-term immune tolerance without causing generalized immunosuppression.

### Senescent cell clearance

Cellular senescence represents a fundamental biological process marked by irreversible cell cycle arrest.^125^ This physiological state is accompanied by profound alterations in cellular architecture and function, including chromatin remodeling, metabolic reprogramming, and the development of a distinctive senescence-associated secretory phenotype (SASP). While serving as an important tumor suppression mechanism, the pathological accumulation of senescent cells contributes significantly to chronic inflammation, tissue dysfunction, and malignant transformation through SASP-mediated bystander effects. The therapeutic potential of senescent cell clearance has been established in preclinical studies, prompting the development of targeted elimination strategies. A key advancement in this field involves the identification of urokinase-type plasminogen activator receptor (uPAR) as a nearly universal surface marker of senescent cells. This discovery has enabled the design of precision CAR-T therapies capable of specifically recognizing and eliminating senescent cell populations. Preclinical evidence demonstrates the efficacy of this approach: uPAR-targeted CAR-T cells effectively reduce senescent cell burden in vivo, treatment extends survival in murine models of lung adenocarcinoma, therapeutic benefits include restoration of tissue homeostasis in liver fibrosis models.^126–130^ These findings highlight the potential of CAR-T technology to address senescence-related pathologies through targeted cell clearance, offering advantages over traditional senolytic approaches by providing: antigen-specific senescent cell recognition, in vivo persistence for sustained effects, capacity for tissue penetration and immune memory against recurring senescent cells.

The success of uPAR-directed CAR-T cells in preclinical models establishes a proof-of-concept for immunologically targeting cellular senescence, with potential applications across age-related diseases, fibrotic disorders, and senescence-associated malignancies.

### Pathogen-specific targeting

CAR-T cell therapy has demonstrated versatility in targeting pathogen-infected cells through specialized receptor designs. This approach leverages pathogen-specific surface markers to enable precise immune recognition and clearance of infected host cells. Several engineered CAR-T cells have shown promising results against diverse infectious agents: HIV-specific bNAbs-CAR-T cells effectively identify and eliminate HIV-infected cells by targeting conserved viral epitopes,^131^ for hepatitis B virus (HBV) infection: S/L-CAR-T cells specifically recognize HBV surface antigens or demonstrating potent activity against HBV-infected hepatocytes,^79,132,133^ in cytomegalovirus (HCMV) infection: gB-specific CAR-T cells target the viral glycoprotein B and show specific cytotoxicity against HCMV-infected cells, and antifungal applications: Dectin-1 CAR-T cells recognize β-glucans on Aspergillus species, CD8^+^ Af-CAR-T cells target fungal surface antigens and both demonstrate effective clearance of fungal pathogens.^50,134,135^ These cells directly act on infected cells to form an immune clearance effect. The development of these targeted therapies represents a significant advancement in antimicrobial immunotherapy, offering potential advantages over conventional treatments through their specificity, adaptability, and capacity for persistent surveillance. Each CAR construct is carefully designed to recognize conserved pathogen structures and minimizing off-target effects.

## Applications by disease category

### Autoimmune diseases

#### Systemic diseases

##### Systemic lupus erythematosus (SLE)

Systemic lupus erythematosus (SLE) is a heterogeneous autoimmune disease driven by autoreactive B cells producing antinuclear antibodies (ANA), which form immune complexes, triggering inflammation and organ damage.^136,137^ Innate immune dysregulation involves excessive type I interferon (IFN) signaling, neutrophil extracellular traps (NETs), and dendritic cell (DC) activation via TLRs, promoting BAFF production and chronic immune activation.^138–140^

Adaptive immune dysfunction includes T-cell abnormalities, leading to aberrant cytokine release and CD40L-mediated B-cell activation, while Treg and Breg impairment disrupts immune tolerance, sustaining autoantibody production and inflammation.^137,141–144^

Significant progress has been made in the treatment of SLE over the past few decades; existing treatment options include corticosteroids, antimalarial drugs, immunosuppressants, and biologics. However, these treatments require long-term use, which can have significant side effects, and their effectiveness often diminishes over time.^145,146^ Current methods for eliminating alloantibodies include plasma exchange, intravenous immunoglobulin, and monoclonal antibodies. However, these approaches cannot eradicate memory B cells or plasma cells, resulting in limited therapeutic efficacy. The demonstrated success of CAR-T cells in B-cell malignancies—where CD19 CAR-T cells have achieved long-term remission in chronic lymphocytic leukemia patients and eliminated tumor cells in acute lymphoblastic leukemia and B-cell non-Hodgkin lymphoma-suggests potential applications for systemic lupus erythematosus (SLE). The primary mechanism of CD19 CAR-T cells involves binding to CD19 on B-cell surfaces, triggering a cascade of immune responses and curing B-ALL. Theoretically, this long-term B-cell depletion mechanism could be adapted for SLE treatment.^147^ In-depth investigation of B-cell depletion mechanisms following CAR-T therapy in SLE patients has confirmed selective CAR T cell-mediated resetting of responsive B-cell populations, while revealing transcriptional changes in monocyte and T-cell subsets—particularly marked reduction in type I IFN signaling that correlates with SLE pathogenesis, providing rationale for CAR-T therapy in SLE. Current clinical trials have progressed to Phase I, demonstrating symptom relief (reduced proteinuria, decreased anti-dsDNA antibody levels) in SLE patients. For instance, Li et al. have found a woman with refractory thrombocytopenia complicating SLE had a response to CD19 CAR T cells with an increase in platelet counts and minimal symptoms of cytokine release syndrome (CRS),^148^ Mackensen et al. treated 5 SLE patients with CD19 CAR-T cells following lymphodepletion,^114^ while Müller et al. treated 8 patients, observing sustained clinical improvement for up to two years with only mild CRS.^149^ Unlike in malignancies, SLE patients showed rapid immune cell recovery post-treatment, suggesting preserved immune competence. A case report by Mougiakakos et al. described rapid resolution of acute symptoms without adverse events in a severely affected 20-year-old SLE patient, confirming that pathogenic autoantibodies originate from CD19^+^ B cells and supporting CD19 CAR-T efficacy.^150^ However, pre-treatment lymphodepletion in these studies may confound accurate assessment of CAR-T effects,^149^ though remains necessary to ensure therapeutic efficacy of adoptive immunotherapy.^151^ Uppin et al. proposed BAFF-targeting CAR-T cells (against BAFF-R, BCMA, and TACI antigens on mature B cells) based on observed clinical improvement with BAFF-specific belimumab and CD20-specific rituximab in SLE patients. Humanized mouse models demonstrated symptom relief with such CAR-T cells, with theoretical advantages of selectively depleting pathogenic mature B cells while sparing immature B cells and CD19- plasma cells.^152^ However, current evidence remains limited to in vitro and mouse studies—significant interspecies differences (e.g., stronger BAFF-BCMA binding in humans) may enhance clinical efficacy, but human SLE heterogeneity necessitates further safety and effectiveness studies. Notably, BCMA expression on mature B cells increases with serum BAFF levels (particularly on CD19^+^ B cells),^153^ and memory B cells/plasma cells resistant to CD19 CAR-T may reactivate to produce autoantibodies.^154^ BAFF CAR-T cells could enable sustained remission if achieving long-term persistence.^152^

Zhang et al. developed combo CD19/BCMA (APRIL) CAR-T cells that effectively cleared B-cell and plasma-cell subpopulations in murine models. In desensitization experiments, combo CAR-T outperformed single-target therapies, reducing donor-specific antibody (DSA) levels below baseline, and suggesting potential to eliminate vaccine-induced protective antibodies and HLA alloantibodies which minimizing graft-versus-host reactions.^155^ This contrasts with CD19 CAR-T’s inability to clear such antibodies.^156^ While BCMA CAR-T alone shows limited efficacy against B cells (allowing plasma-cell repopulation),^157^ combining with Treg CAR-T may maintain therapeutic effects through immune modulation.^157^

Wang et al. initiated Phase I trials of combo CAR-T in SLE patients, demonstrating effective pathogenic cell clearance, immune reset, and symptom improvement with good safety—particularly promising for refractory cases.^115^ However, long-term persistence data (inferred from depletion levels) lack clonal tracking post-treatment. The impact on other antibody types (e.g., anti-MHC I/II or drug antibodies) remains unaddressed—particularly relevant given SLE’s diverse autoantibody profile beyond just antinuclear antibodies.^157^ The challenge of long-lived plasma cells (LLPCs) lacking surface immunoglobulin expression requires mechanistic investigation to improve targeting, as this underlies treatment resistance in late-stage SLE. CAR-T’s antigen-specific precision could prove transformative if surface markers on pathogenic plasma-cell subsets can be identified.

**Fig. 3:**
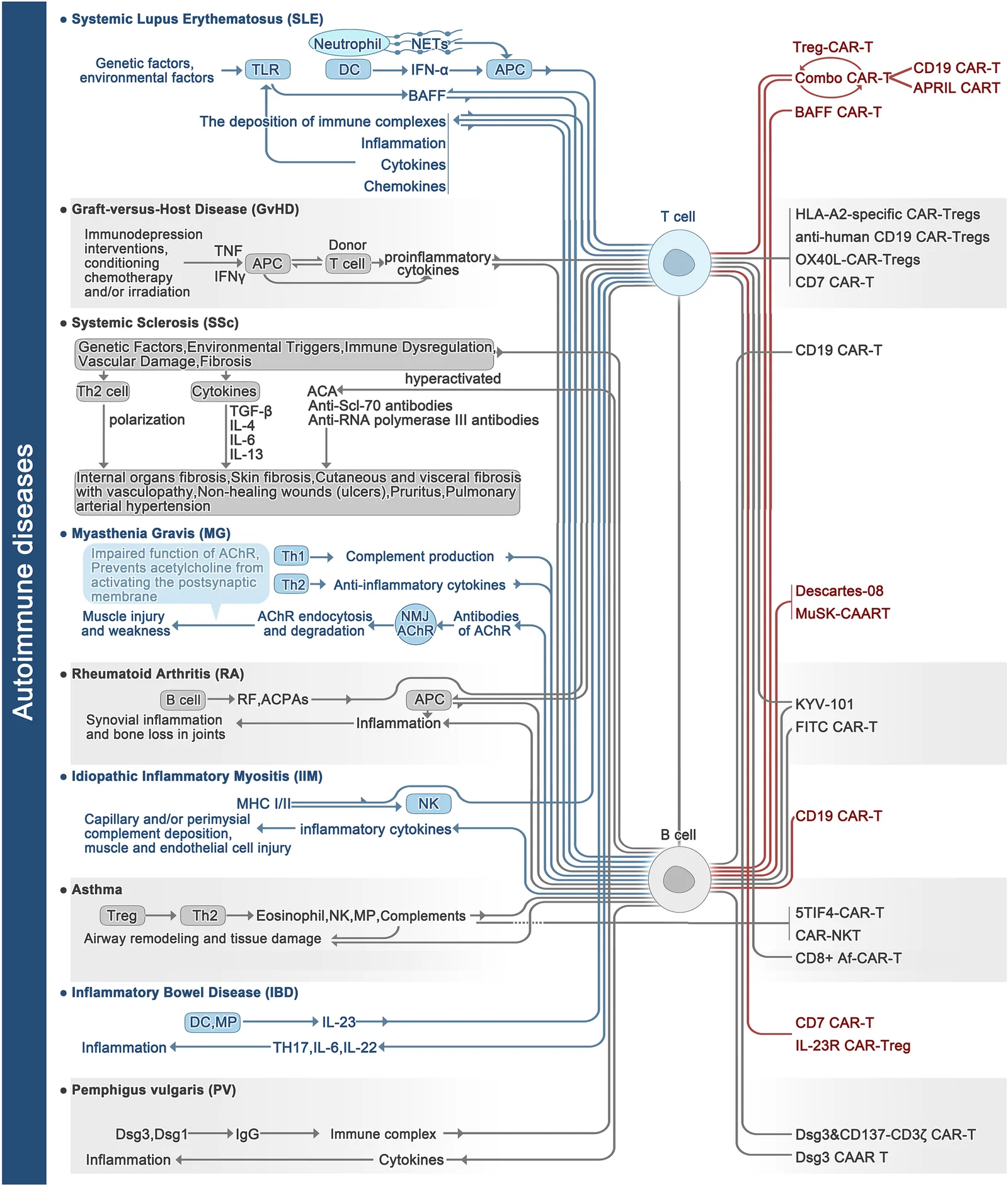
Mechanisms of autoimmune diseases. 1, Systemic Lupus Erythematosus (SLE) is triggered by environmental and genetic factors, leading to immune dysregulation. This manifests as excessive formation of neutrophil extracellular traps (NETs), abnormal expression of the type I interferon gene phenotype causing over-secretion, and activation of Toll-like receptors (TLRs). These processes induce dendritic cell (DC) activation and the production of B cell-activating factors (BAFF), creating a positive feedback loop between these factors and cells. This ultimately leads to B cell activation and the production of autoantibodies. These autoantibodies form immune complexes that abnormally deposit in tissues and are presented by antigen-presenting cells to T cells, which due to inherent dysfunction are activated alongside B cells, leading to tissue damage. CAR-T therapies such as BAFF (targeted to BAFFR and BCMA) CAR-T cells, CD19 CAR-T cells, and Combo CAR-T cells can recognize and eliminate the autoantibody-producing B cells. CAR-Treg help regulate the immune environment and support the function of BCMA CAR-T cells. 2, Graft-versus-host disease (GVHD) typically arises from host tissue damage caused by radiation or chemotherapy. Damage-associated molecules like TNF and type I interferons are recognized by host antigen-presenting cells (APCs), which activate donor T cells, triggering a cascade of immune-mediated damage. HLA-A2-specific CAR-Tregs, anti-human CD19 CAR-Tregs, and OX40L-CAR-Tregs are used to suppress excessive T cell responses. CD7 CAR-T cells eliminate CD3⁺, CD7⁺ T cells and NK cells. 3, Systemic sclerosis (SSc) is a complex autoimmune disease with a multifactorial etiology involving genetic predisposition, environmental triggers, immune dysregulation, vascular damage, and fibrosis. The primary driver is immune dysregulation, characterized by Th2 cell polarization and hyperactivated B cells; B cells produce anti-centromere antibodies (ACA), anti-Scl-70 antibodies, and anti-RNA polymerase III antibodies. Dysregulated cytokines, including TGF-β, IL-4, IL-6, and IL-13, contribute to excessive pro-fibrotic signaling. These pathological processes lead to fibrosis in internal organs and skin, vasculopathy, non-healing wounds (ulcers), pruritus, and complications such as pulmonary arterial hypertension (PAH). CD19 CAR-T cell targets and eliminates pathogenic B cells, this approach has shown potential in reversing fibrosis and restoring immune balance. 4, Myasthenia Gravis involves Th1 and Th2 cells causing direct damage to acetylcholine receptors (AChRs) under the effect of anti-AChR autoantibodies, along with BAFF-mediated B cell activation, leading to autoantibody production. These mechanisms collectively damage AChRs at the neuromuscular junction, resulting in muscle weakness. Descartes-08 (anti-BCMA RNA CAR-T cells) and MuSK-CAART specifically target and eliminate pathogenic B cells producing these antibodies. 5, Rheumatoid Arthritis (RA) is characterized by autoantibodies such as rheumatoid factor (RF) and anti-citrullinated protein antibodies (ACPAs). These antibodies interact with T cells, recognize APCs or bind to antigens on B cells, triggering cell-to-cell interactions (e.g., inflammation), which cause joint damage and functional impairment. KYV-101 and FITC CAR-T cells are used to target and eliminate autoreactive B cells. 6, Idiopathic Inflammatory Myopathies show elevated expression of MHC class I and II molecules in muscle tissue, which activate NK cells and T cells, leading to direct muscle damage or cytokine production. Complement activation causes immune complex deposition, damaging perimysial blood vessels and muscle cells. CD19 CAR-T cells are used to target and eliminate pathogenic B cells. 7, Asthma is associated with a Th2-mediated immune response, where T cells secrete cytokines (inflammatory mediators). These cytokines activate antibody-dependent cytotoxic cells (neutrophils, macrophages, NK cells) and the complement pathway, resulting in local inflammation or direct tissue damage. In asthma, Treg function is often impaired, reducing their ability to regulate Th2 responses. 5T_IF_4-CAR-T cells target and eliminate eosinophils. Interactions with CAR-NKT cells disrupt allergen–mast cell/basophil interactions. CD8⁺ Af-CAR-T cells target fungal infections that trigger asthma, eliminating the associated T cells. 8, Inflammatory Bowel Disease (IBD) is driven by overproduction of IL-23 by macrophages and dendritic cells, which activates Th17 cells. The resulting cytokines cause local inflammation and recruit additional immune cells via positive feedback. CD7 CAR-T cells target and eliminate pathogenic T cells, while IL-23R CAR-Tregs and regulatory CD4⁺CD25⁺FoxP3⁺ T cells (Tregs) modulate the immune response and reduce levels of IFN-γ and TNF. 9, Pemphigus Vulgaris is primarily caused by IgG autoantibodies against desmoglein (Dsg) core proteins in desmosomes. B cells that produce these antibodies form immune complexes, activate immune cells, and induce cytokine release, recruiting immune cells and promoting inflammation and memory B cell formation, leading to recurrent inflammation. Dsg3 CAAR-T cells specifically target B/plasma cells expressing Dsg3-specific B cell receptors. CAR-T cells expressing a recombinant Dsg3–CD137–CD3ζ T cell receptor eliminate these pathogenic cells. BCMA B-cell maturation antigen, NMJ neuromuscular junction, MP/Mø macrophages

CAR-T cells applied to the treatment of systemic lupus erythematosus (SLE) still face several challenges, such as: differences in B cell kinetics between SLE and leukemia patients: B cell reconstitution in SLE patients occurs more rapidly than in leukemia patients, so the kinetics of CD19 CAR-T cells in SLE need further exploration, particularly regarding their targeting efficiency and persistence.^114,115,158^ B cell regeneration impairment: CAR-T therapies targeting B cell lineage markers (such as CD19) or B cell maturation antigen (BCMA) have shown efficacy but have chance to lead to long-term B cell depletion.^159,160^ To improve specificity, chimeric autoantibody receptor T (CAAR-T) cells have been developed to selectively eliminate autoreactive B cells while sparing healthy ones. However, CAAR-T therapy is limited in its long-term effectiveness due to its inability to effectively target long-lived plasma cells.^161^ Target selection challenges: While 100% of SLE patients test positive for antinuclear antibodies (ANA), the presence of other autoantibodies varies. Identifying the optimal autoantibody target for CAR-T therapy remains a critical and unresolved challenge^162^ (Fig.3).

Currently, there are numerous clinical trials targeting SLE, most of which are still in Phase I and primarily focused on assessing adverse drug reactions. The promising early-phase trial data position CAR-T therapy as a potential paradigm shift in SLE treatment. However, addressing SLE’s profound heterogeneity remains the central challenge. The varying autoantibody profiles among patients question the viability of universal CAR-T products and point toward future personalized targeting strategies. Beyond initial B-cell depletion, the field is advancing toward precision approaches like CAAR-T cells. Yet the persistent barrier of long-lived plasma cells (LLPCs)—resistant to current therapies due to lack of surface targets—demands novel antigen discovery or combinatorial strategies. Notably, the rapid immune reconstitution and sustained remission observed in early trials suggest CAR-T may enable an “immune reset” in autoimmune contexts, distinct from its oncological mechanism. Future success hinges on long-term clonal tracking of CAR-T cells and detailed monitoring of the reconstituted immune repertoire to verify durability. Ultimately, transitioning CAR-T therapy into clinical practice will require trials that stratify patients by immunopathological signatures, moving toward truly personalized cellular therapy for autoimmune disease.

##### Graft-versus-host disease (GvHD)

Graft-versus-host disease (GvHD) is a condition that occurs following allogeneic hematopoietic stem cell transplantation (allo-HSCT). It arises when the surviving allogeneic immune effector cells in the recipient’s body attack the recipient’s organs, leading to complications in various tissues. On a microscopic level, GvHD is triggered when donor T cells encounter allogeneic stimuli, become activated, secrete cytokines, and proliferate into effector cells. This initial donor anti-host response induces a systemic effect that manifests as a multi-organ syndrome. There are two types of GvHD: acute GvHD(aGvHD) and chronic GvHD(cGvHD). Acute GvHD progresses through three phases: tissue damage from conditioning releases DAMPs/PAMPs, activating host APCs; donor CD4^+^/CD8^+^ T cells are primed by APCs; and effector T cells and cytokines (e.g., TNF, IFN-γ) target epithelial cells, causing apoptosis and organ damage.^163^ Chronic GvHD develops through overlapping stages: early inflammation, thymic injury leading to immune dysregulation (escape of autoreactive T cells), and fibrosis driven by IFN-γ and cytokine imbalance (e.g., IL-6, TNF-α). Unlike aGvHD, cGvHD involves both donor alloreactive and host autoreactive T cells, perpetuating tissue fibrosis.^164,165^

T cells are the primary pathogenic factor. The broad expression of CD7 on T cells and their precursors provides a therapeutic target.^166^ Li et al. reported a case study where a patient received universal CD7 CAR-T cell therapy after chemotherapy preconditioning, resulting in complete elimination of tumor cells. The therapy effectively expanded CD7 CAR-T cells and eradicated CD3^+^, CD7^+^ T cells and natural killer (NK) cells, with therapeutic effects lasting up to four months. The patient subsequently underwent hematopoietic stem cell transplantation to restore hematopoiesis and remained free of graft-versus-host disease (GvHD) for three months without immunosuppressive drugs.^167^ This suggests that universal CD7 CAR-T therapy may potentially reduce GvHD risk while creating a favorable environment for transplantation. However, other studies have reported GvHD occurrence following universal CD7 CAR-T cell therapy.^167^ Therefore, the efficacy of universal CD7 CAR-T against GvHD remains uncertain and requires further investigation. Key questions include whether prolonged CD7^+^ T cell depletion may lead to post-transplant relapse and/or increased infection risk, and whether CD7^-^ T cells retain graft-versus-leukemia (GVL) effects and anti-infection capabilities.^166,168^

Regulatory T cells (Tregs) show promising potential for GvHD treatment by preventing or mitigating unwanted immune responses in transplantation or autoimmune conditions.^169^ Recent advances demonstrate that HLA-A2-specific CAR-Treg cells can effectively suppress HLA-A2^+^ peripheral blood mononuclear cell (PBMC) proliferation in vitro and prevent xenogeneic GvHD induced by HLA-A2^+^ PBMCs in mice.^170^ Dawson et al. also observed HLA-A2^+^ cell-mediated rejection and accordingly developed a humanized Treg cell product (hA2-CAR Tregs) that reduced graft rejection, providing supportive evidence for clinical application of engineered Tregs.^31^ Additionally, anti-human CD19 CAR-Tregs have been shown to suppress antibody production in immunodeficient mice reconstituted with human PBMCs, achieving GvHD inhibition without compromising graft-versus-tumor (GVT) effects.^171^

While these findings strongly support Treg applications, it should be noted that CAR-T cell therapy itself may induce GvHD. Consequently, managing CAR-T-induced GvHD has gained attention, primarily through genetic engineering approaches. One strategy involves TCR knockout (particularly TRAC gene disruption) to prevent T cell recognition and attack of host tissues, such as utilizing non-αβ T cells or genetically eliminating TCR from αβ T cells.^172^ Another approach incorporates an inducible caspase-9 (iCasp9) suicide gene system, enabling pharmacological induction of apoptosis in case of severe toxicity or off-target effects.^173^ The safety profile of CAR-T cell therapy for GvHD requires further validation. Given the high costs of autologous T cell modification, allogeneic CAR-T cell research is actively progressing. Alternative strategies include (Fig. 3).

CAR-T cells therapy for GvHD is evolving along two complementary paths: universal CAR-T cells targeting markers like CD7 aim to eliminate pathogenic immune cells, while engineered Tregs focus on restoring immune tolerance. Despite promising early results, key challenges remain. Preserving graft-versus-leukemia effects and anti-infection capacity during targeted T-cell depletion requires careful balance. Additionally, while genetic engineering strategies such as TCR knockout and suicide switches have improved the safety profile of allogeneic CAR-T products, concerns regarding delayed immune reconstitution and long-term immunosuppression persist. Looking ahead, the field should prioritize developing precision-targeting strategies, including bispecific CARs or conditionally activated systems that distinguish pathogenic from protective immune cells. Combining engineered Tregs with targeted depletion therapies may establish a dual-layer “suppression-and-clearance” network for sustained tolerance. Furthermore, smart CAR-T cells capable of dynamically responding to biomarkers could pave the way for personalized GvHD treatment. As our understanding of GvHD pathogenesis deepens and gene-editing technologies advance, cell therapy holds potential to transition from an adjunct intervention to a curative modality for GvHD.

##### Systemic sclerosis (SSc)

Systemic sclerosis (SSc) is a complex autoimmune disease characterized by widespread organ fibrosis. Clinical manifestations include mobility impairment, non-healing wounds (ulcers), pruritus, pulmonary arterial hypertension, and organ failure, among others. Current conventional treatments for SSc primarily involve low-dose glucocorticoids combined with immunosuppressants such as cyclophosphamide and azathioprine. For patients with comorbid interstitial pneumonia, antifibrotic agents like pirfenidone or nintedanib are often used. Some patients may benefit from biologic therapies such as rituximab. However, overall, the efficacy of currently available treatments for SSc remains limited, often only slowing disease progression. Existing therapies cannot reverse established organ fibrosis, and some patients show poor response to all available drugs, resulting in an unfavorable prognosis.^174^

Recent studies suggest that B cells play a crucial role in the pathogenesis of SSc, contributing to immune dysregulation, fibrosis, and vascular dysfunction. B cells secrete large quantities of autoantibodies and promote the release of pro-inflammatory or pro-fibrotic cytokines (particularly IL-6), interacting with other SSc-related cells such as vascular endothelial cells, vascular smooth muscle cells, and fibroblasts. This leads to extracellular matrix deposition, ultimately causing fibrosis in the skin, muscles, and internal organs.^175,176^ In a groundbreaking study, Schett et al. first employed anti-CD19 CAR-T cell therapy to treat a severely refractory SSc patient. The patient, a 60-year-old male, exhibited severe diffuse myocardial fibrosis, pulmonary fibrosis with pulmonary hypertension, Raynaud’s phenomenon, and wrist arthritis, with no response to immunosuppressive therapy. Following infusion of CAR-T cells (1 × 10^6^ cells/kg), the patient showed improvement in skin fibrosis and resolution of wrist arthritis. Both the EUSTAR AI activity score and the modified Rodnan Skin Score (mRSS) decreased. The patient also reported reduced frequency and severity of Raynaud’s episodes.^177^

Subsequently, the team enrolled four additional refractory SSc patients in a clinical trial. After CD19 CAR-T cell infusion, analysis of SSc-related autoantibodies revealed a moderate reduction in anti-Scl-70 antibodies, while autoantibodies targeting RP11, RP155, PM-Scl70, and CENP-B disappeared post-treatment. Correspondingly, these patients exhibited significant reductions in EUSTAR AI activity scores, mRSS, glucocorticoid dependence, and the need for immunosuppressive therapy.^149^

Serial studies observed the re-emergence of naive, non-class-switched B cells, the disappearance of circulating plasmablasts, and downregulation of disease-associated immunoglobulin heavy and light chains, supporting the concept that CD19 CAR-T cell therapy may induce a “reset” of pathological autoimmunity in these patients. Although CAR-T cells target both pathogenic and non-pathogenic B cells, the reconstitution of the B-cell compartment after CAR-T cell depletion appears to occur in the absence of autoreactive B-cell clones, potentially explaining long-term disease remission. However, the contribution of concomitant immunosuppressive therapy to the short-term effects of CAR-T treatment cannot be entirely ruled out, necessitating further observation of drug-free disease progression in SSc. Given the limited immunosuppressive intensity of the conditioning regimen (lower than standard SSc treatment protocols), additional studies involving more patients—particularly those with poor prognostic factors such as anti-Scl-70 positivity—are warranted.

In another study, Merkt et al. utilized third-generation CD19 CAR-T cells to treat a 38-year-old woman with anti-Scl-70-positive SSc and rapidly progressive nonspecific interstitial pneumonia (NSIP). The addition of CD19 CAR-T cells to her existing mycophenolate/nintedanib regimen led to sustained improvement in pulmonary function, accompanied by significant radiological regression. This study was the first to describe the disappearance of Fc receptor-activating immune complexes in an autoimmune disease following CAR-T therapy. In SSc, such immune complexes are thought to contribute to pathology via Fcγ receptor activation. This innovative approach improved pulmonary outcomes and optimized immunosuppressive therapy, though the underlying mechanisms require further investigation. Additionally, whether the persistence of CAR-T cells plays a deterministic role remains to be validated in larger cohorts^178^ (Fig. 3).

CD19 CAR-T therapy shows promising results in refractory systemic sclerosis, demonstrating improvement in skin fibrosis, arthritis, and pulmonary function with reduced clinical scores. The treatment appears to achieve immunological reset through B-cell depletion and reconstitution, evidenced by plasmablast disappearance and autoantibody profile improvement without autoreactive clone recurrence. However, key questions require further investigation: the contribution of concomitant immunosuppression to therapeutic outcomes needs clarification, particularly given the mild preconditioning regimen. The mechanistic relationship between Fc receptor-activating immune complex disappearance and clinical improvement warrants exploration. Additionally, the correlation between CAR-T persistence and long-term efficacy remains unverified. Addressing these issues through larger studies, especially in high-risk populations, will be crucial for standardizing and optimizing this innovative approach for SSc treatment.

#### Musculoskeletal system

##### Myasthenia gravis (MG)

Myasthenia gravis (MG) is an autoimmune disorder where antibodies target nicotinic acetylcholine receptors (AChRs) at neuromuscular junctions, impairing synaptic transmission and causing muscle weakness via complement activation, AChR blockade, and accelerated degradation.^179–181^ CD4^+^ T helper cells (Th1, Th2, Th17) drive pathogenic anti-AChR antibody production through pro-inflammatory cytokines (IFN-γ, IL-17A) and B-cell activation, while Tregs (CD4^+^CD25^+^FOXP3^+^) and Th3 cells (TGF-β) regulate immune tolerance.^182–186^ A rare MuSK-MG subtype involves anti-MuSK antibodies, which disrupt AChR clustering and synaptic folding, distinct from AChR-MG pathogenesis.^187^

Anti-BCMA RNA CAR-T cells (rCAR-T) called Descartes-08 are being explored for treating autoimmune diseases.^188,189^ In MG, muscle-specific tyrosine kinase (MuSK) antibodies, primarily IgG4, disrupt the physiological functions of MuSK in synaptic maintenance and adaptation, causing disintegration of the neuromuscular junction. MuSK is a transmembrane tyrosine kinase with an extracellular domain that includes three immunoglobulin-like (Ig1-Ig3) and frizzled-like (Fz) domains. Studies show that sera from MuSK MG patients can recognize Ig1, Ig2, and Ig3-Fz domains at rates of 100%, 58%, and 23%, respectively.^190^ Researchers have designed T cells to express MuSK-chimeric autoantibody receptor (MuSK-CAART) with a CD137-CD3ζ signaling domain to specifically target B cells that express anti-MuSK antibodies. MuSK-CAART has demonstrated efficacy similar to anti-CD19 CAR-T cells in eliminating anti-MuSK B cells and has retained cytolytic activity even in the presence of soluble anti-MuSK antibodies. The designed MuSK-CAAR structure, which contains the full extracellular domain of MuSK linked to CD137-CD3ζ co-stimulatory and activation domains, has shown comparable activity, indicating that it can be successfully expressed. MuSK-CAART treatment depletes antigen-specific IgG without depleting all B cells and achieves therapeutic effects without prior lymphodepletion. Current research demonstrates promising targeted approaches for myasthenia gravis treatment. Descartes-08, an anti-BCMA RNA CAR-T cell therapy, is under investigation for autoimmune applications. More notably, MuSK-CAART has been engineered to specifically eliminate anti-MuSK antibody-producing B cells while sparing normal B cells. This approach shows comparable efficacy to anti-CD19 CAR-T in depleting target cells, maintains activity despite soluble antibodies, achieves therapeutic effects without lymphodepletion, and has demonstrated no off-target toxicity in preclinical studies. However, these findings remain at the animal testing stage, requiring validation through clinical trials before clinical application can be considered^187^ (Fig. 3).

##### Rheumatoid arthritis (RA)

Rheumatoid arthritis (RA) is a chronic autoimmune disease characterized by progressive joint inflammation and destruction, driven by dysregulated immune responses involving T/B lymphocytes, macrophages, and inflammatory cytokines.^191–195^ Key mechanisms include: T-cell activation by citrullinated antigens via HLA-DRB1, triggering synovitis and anti-citrullinated protein antibody (ACPA) production^196^; macrophage polarization toward proinflammatory M1 phenotypes (TNF-α, IL-6, IL-1β) with impaired M2-mediated repair^196,197^; and B-cell dysfunction, producing autoantibodies (rheumatoid factor, anti-CCP) and proinflammatory cytokines while regulatory B/T cells (Tregs, Bregs) fail to suppress autoimmunity.^198–203^

Current treatment options include rituximab, an anti-CD20 monoclonal antibody that demonstrates clinical efficacy by depleting B cells.^204,205^ However, this broad depletion strategy carries significant risks of infection and compromises protective immune memory responses.^206,207^ More targeted approaches using CAR-T cell therapy are emerging as potential alternatives. The fully humanized CD19-CAR-T product KYV-101, previously effective in neurologic autoimmune conditions, has shown promise in inducing remission in RA patients following lymphodepleting chemotherapy,^118^ though the required immunosuppression remains a limiting factor.^208^ Li et al. applied fourth-generation CAR-T cell therapy to three rheumatoid arthritis (RA) patients, innovatively integrating three functional modalities: tumor necrosis factor (TNF) inhibition, IL-6 suppression, and B-cell depletion. This approach not only eliminates B cells but also provides localized anti-inflammatory effects by targeting cytokine networks at B-cell infiltration sites in RA joints. The study demonstrated: robust in vivo expansion of CAR-T cells, complete eradication of CD19^+^ B cells, clearance of RA-associated autoantibodies, and sustained high-level clinical responses with persistent CAR-T cell activity.^209^ Another innovative approach utilizes universal FITC-CAR-T cells designed to target FITC-labeled RA-specific peptides, which have demonstrated dose-dependent cytotoxicity against autoreactive B cell subsets in vitro. While these CAR-T strategies show encouraging preliminary results, their application in RA remains largely conceptual, awaiting validation through preclinical in vivo studies and clinical trials.^210,211^ Future progress in autoantigen discovery and multi-omics technologies (combining serological, proteomic, and genetic analyses) may enable the development of precisely targeted CAR-T designs capable of selectively eliminating pathogenic lymphocyte populations while preserving protective immunity, building on current experimental successes in this rapidly evolving field (Fig. 3).

KYV-101 shows clinical potential despite requiring immunosuppressive preconditioning. More innovative designs include fourth-generation CAR-T cells combining B-cell depletion with localized TNF and IL-6 suppression, demonstrating multi-modal efficacy in early studies. Additionally, universal FITC-CAR-T platforms enable targeting of specific autoreactive B-cell subsets. While these approaches present promising alternatives to conventional therapies like rituximab, they remain primarily conceptual for RA treatment. Further validation through preclinical in vivo studies and clinical trials is essential to establish their therapeutic potential and safety profile in rheumatoid arthritis.

##### Idiopathic inflammatory myositis (IIM)

Idiopathic inflammatory myositis (IIM) comprises six autoimmune subtypes characterized by muscle weakness and cutaneous manifestations, classified by myositis-specific autoantibodies.^207,211^ Key pathogenic mechanisms include: MHC I/II upregulation on muscle tissue, activating T/NK cells and causing myofiber damage; complement deposition in capillaries (dermatomyositis (DM), polymyositis (PM), anti-synthetase syndrome (ASS), immune-mediated necrotizing myopathy (IMNM)) and proinflammatory cytokines (IL-1, TNF, interferons) amplifying inflammation; and TREM-1-mediated immune cell migration and TNF-α secretion.^207,210–214^ Distinct pathological mechanisms operate across different IIM subtypes. In IMNM, autoantibodies directly contribute to myotube and muscle fiber destruction,^215^ while ASS is characterized by autoantibodies targeting aminoacyl-tRNA synthetases, with antibody titers often correlating with clinical disease severity.^216^

Emerging clinical evidence demonstrates the therapeutic potential of CD19-targeted CAR-T cell therapy in refractory cases. Two male ASS patients who had failed multiple conventional immunosuppressive therapies, including rituximab and intravenous immunoglobulin, showed rapid and sustained clinical improvement following CAR-T treatment.^217,218^ Both patients achieved seroconversion of anti-Jo-1 antibodies, normalization of creatine kinase levels, and significant improvements in muscle strength and respiratory function. Similar outcomes were observed in a 45-year-old female patient with refractory myositis, arthritis, and pulmonary involvement who had previously failed nine different treatment regimens. Following CD19 CAR-T therapy, she demonstrated marked improvement across all disease manifestations according to ACR/EULAR criteria and maintained drug-free remission during 150 days of follow-up.^219^ Additional case reports have corroborated these findings, showing consistent efficacy of CD19 CAR-T therapy in ASS patients.^176,219,220^ While these clinical observations are promising, several key questions remain unanswered. The precise mechanism by which CD19-targeted CAR-T cells ameliorate IIM symptoms requires further elucidation, particularly given that some patients received combination therapies making it challenging to isolate the specific contribution of CAR-T treatment.^206,221,222^ Current research efforts include an ongoing Phase I/II clinical trial (NCT06154252) focused on evaluating adverse events and treatment efficacy^223^ (Fig. 3).

CD19-directed CAR-T cell therapy demonstrates promising clinical potential in refractory idiopathic inflammatory myopathies, particularly in anti-synthetase syndrome (ASS). Multiple case reports document rapid and sustained improvement in muscle strength, respiratory function, and serological markers following treatment, with some patients achieving drug-free remission. However, the precise therapeutic mechanism remains unclear, complicated by concurrent immunosuppressive therapies in reported cases. Future research should focus on elucidating mechanisms of action, optimizing CAR constructs, establishing patient selection criteria, and conducting long-term safety monitoring to fully assess this innovative therapeutic approach.

#### Respiratory system

##### Asthma

Asthma is a chronic respiratory disease affecting over 300 million people worldwide, characterized by airway inflammation, hyperresponsiveness (AHR), and reversible airflow obstruction.^224–227^ The pathophysiology of allergic asthma involves: epithelial alarmins (IL-25, IL-33, TSLP) activating ILC2s and Th2 cells, driving IL-4/5/9/13 production, eosinophilia, and IgE-mediated inflammation.^228–230^ And Treg suppression via CTLA-4 and granzyme-perforin mechanisms to balance Th2 responses.^231–234^ Current therapies (steroids, biologics) face limitations, prompting exploration of CAR-T cells for durable immunomodulation.^235–238^

Innovative CAR-T strategies are being developed to precisely target these pathogenic pathways. A breakthrough approach involves engineered 5T_IF_4 cells—IL-5Rα-targeting CAR-T cells with BCOR/ZC3H12A knockout that co-express an IL-4 mutein to simultaneously block IL-4/IL-13 signaling.^35,239^ This design achieved durable suppression of allergic asthma in murine models with a single administration, eliminating the need for chronic treatment or lymphodepleting preconditioning. Alternative strategies leverage NKT cells’ unique immunomodulatory properties, with CAR-NKT constructs targeting IgE receptors to disrupt allergen–mast cell interactions while secreting anti-inflammatory cytokines (IFN-γ, TGF-β, IL-10).^240–247^ For IgE-driven disease, EMPD-specific CAR-T cells selectively deplete membrane IgE^+^ B cells without affecting circulating IgE or non-IgE producing B cell subsets, demonstrating precise targeting in preclinical models.^248–250^ Fungal asthma presents distinct challenges being addressed through dectin-1 CAR constructs that recognize β-glucans on Aspergillus and other fungi, with CD8^+^ Af-CAR-T cells showing potent antifungal activity via granzyme B and perforin release.^50,251–257^ Despite these promising developments, significant hurdles remain for clinical translation. The requirement for lymphodepleting chemotherapy poses challenges in non-oncological diseases like asthma, where the toxicity-risk profile must be exceptionally favorable.^35^ Additional concerns include potential on-target/off-tissue effects given shared antigen expression between pathogenic and normal cells,^258^ risks of immune surveillance impairment from prolonged B cell depletion, and theoretical oncogenic potential from genetic modifications.^230,249,259,260^ Most current evidence derives from murine models, underscoring the need for rigorous preclinical optimization and clinical validation to establish safety and efficacy profiles suitable for chronic respiratory conditions. Future directions will need to address these challenges while refining cellular persistence, specificity, and controllability to realize the potential of CAR-based therapies for asthma and other allergic diseases (Fig. 3).

#### Digestive system

##### Inflammatory bowel disease (IBD)

Inflammatory bowel disease (IBD) arises from disrupted intestinal barrier function and dysregulated immune responses, creating a vicious cycle of inflammation.^261–269^ Key mechanisms include: IL-23/Th17 axis activation (via dendritic cells/macrophages) driving IL-17A/F and IL-22 production, amplifying T-cell-mediated inflammation^270,271^; and pathogenic B-cell involvement, where mucosal B cells and plasma cells in refractory cases produce pro-inflammatory cytokines and autoantibodies against epithelial cells, exacerbating tissue damage and treatment resistance.^272^

Novel CAR-T cell strategies are being explored to target these pathogenic mechanisms. CD7-directed CAR-T cells offer potential by eliminating specific immune subsets (T cells, NK cells) involved in intestinal inflammation, as CD7 is expressed on these cell surfaces.^273^ An alternative approach utilizes engineered IL-23R CAR-Tregs, where regulatory T cells expressing the IL-23 receptor chimeric antigen receptor can suppress mucosal inflammation. These modified Tregs have demonstrated therapeutic potential in colitis models by reducing proinflammatory cytokine production (IFN-γ, TNF) while maintaining their immunoregulatory functions.^274,275^ However, IBD therapy development faces several challenges. The disease’s complex etiology complicates target selection, with current research primarily focusing on universal CD7 CAR-T cells and regulatory T cell approaches.^273^ Notably, observations from oncology research reveal that CD19 CAR-T cells can accumulate in intestinal tissue, inducing IBD-like symptoms.^276^ This phenomenon warrants careful consideration for two reasons: it may inform strategies to mitigate gastrointestinal toxicity in lymphoma treatment, while simultaneously providing insights into IBD pathogenesis through the study of CAR-T cell interactions with intestinal mucosa. These findings also highlight potential safety concerns regarding CAR-T cell localization and activation patterns in non-malignant conditions. As the field progresses, further investigation is needed to elucidate optimal cellular targets, improve specificity to avoid off-tissue effects, and develop strategies to control CAR-T cell trafficking and persistence. The dual potential of CAR-T therapies—to either treat or paradoxically induce intestinal inflammation—underscores the importance of rigorous preclinical evaluation and careful clinical translation for IBD applications (Fig. 3).

#### Skin system

##### Pemphigus vulgaris (PV)

Pemphigus vulgaris (PV) is a severe autoimmune blistering disorder caused by IgG autoantibodies against desmoglein, leading to skin/mucosal blistering and increased mortality.^277^ Key pathogenic mechanisms include: CD4^+^ T cell-dependent activation of autoreactive B cells, generating Dsg-specific IgG through IL-5/6, TNF-α, and SDF-1α-mediated inflammation^278–281^; and Autoantibody-mediated disruption of epidermal adhesion, with IgG deposition directly causing acantholysis. Current therapies (corticosteroids or/and immunosuppressants) improve outcomes but carry significant risks (infections or cancer),^282–288^ underscoring the need for targeted treatments.

Research on autoantibody production has opened new avenues for CAR-T cell therapy applications. Ellebrecht et al. developed Dsg3 CAAR-T cells by fusing the PV autoantigen with CD137-CD3ζ, which showed specific cytotoxicity against Dsg3-expressing B cells/plasma cells. In mouse models where experimental pemphigus was induced through Dsg3 hybridoma cell transfer, administration of Dsg3 CAAR-T cells prevented disease development.^289^ These engineered cells demonstrate targeted cytotoxicity against PV autoantigen-presenting cells in vitro, even in the presence of circulating autoantibodies, while maintaining minimal toxicity.^289,290^ Ongoing work by the same group aims to extend this approach to other pemphigus autoantigens like Dsg1,^291^ with related clinical trials currently underway. Therapeutic strategies targeting antibody-secreting B cells offer precise elimination of long-lived plasma cells, potentially enabling long-term remission. However, while CAAR-T cell therapy provides specific targeting of a B-cell populations, its effectiveness against short-lived plasma cells is limited by their lack of target antigen expression. Consequently, these cells can only be cleared indirectly, potentially increasing relapse risk.^292^ Further research is needed to address challenges posed by short-lived plasma cells, particularly given the current limitations in clinical trial data. Additionally, investigation of other potential therapeutic targets requires continued advancement to improve treatment outcomes (Fig. 3).

#### Neurological diseases

##### Multiple sclerosis (MS)

Multiple sclerosis (MS) is a chronic inflammatory disorder driven by an aberrant immune response against myelin protein peptides.^11^ The disease is initiated when peripherally activated CD4^+^ T cells migrate into the central nervous system (CNS), where they are reactivated by antigen-presenting cells (APCs) and recruit additional immune cells, including T cells and macrophages, to form inflammatory lesions. Th1 cells secrete pro-inflammatory cytokines such as IFN-γ and TNF-α, while Th17 cells produce IL-17, IL-21, and IL-22. CD8^+^ T cells further contribute to CNS inflammation by releasing IL-17 and IFN-γ, which activate resident immune cells (microglia, astrocytes, and macrophages) and sustain local inflammatory damage.^293–295^ A key feature of MS is the dysfunction of Foxp3^+^ regulatory T cells (Tregs), which exhibit impaired suppressive activity compared to those in healthy individuals. This deficiency allows autoreactive T cells and activated B cells to drive myelin destruction through cytokine production and autoantibody release.^296–298^ Studies suggest that induced Tregs (iTregs) play a critical role in suppressing pathogenic immune responses in MS, and their dysfunction exacerbates disease progression.^299^ Promisingly, myelin oligodendrocyte glycoprotein (MOG)-specific CAR-Tregs have demonstrated therapeutic potential in experimental autoimmune encephalomyelitis (EAE), a mouse model of MS. CARαMOG-Foxp3-expressing iTregs effectively suppressed T cell responses, particularly in the presence of MOG antigen and macrophages. Brain imaging revealed preferential localization of these engineered Tregs in regions such as the granular layer, lateral septal nucleus, and piriform cortex. Mice treated with CARαMOG-Foxp3 Tregs exhibited reduced CNS levels of IFN-γ and IL-12 compared to controls, highlighting their anti-inflammatory effects.^116,300^

**Fig. 4:**
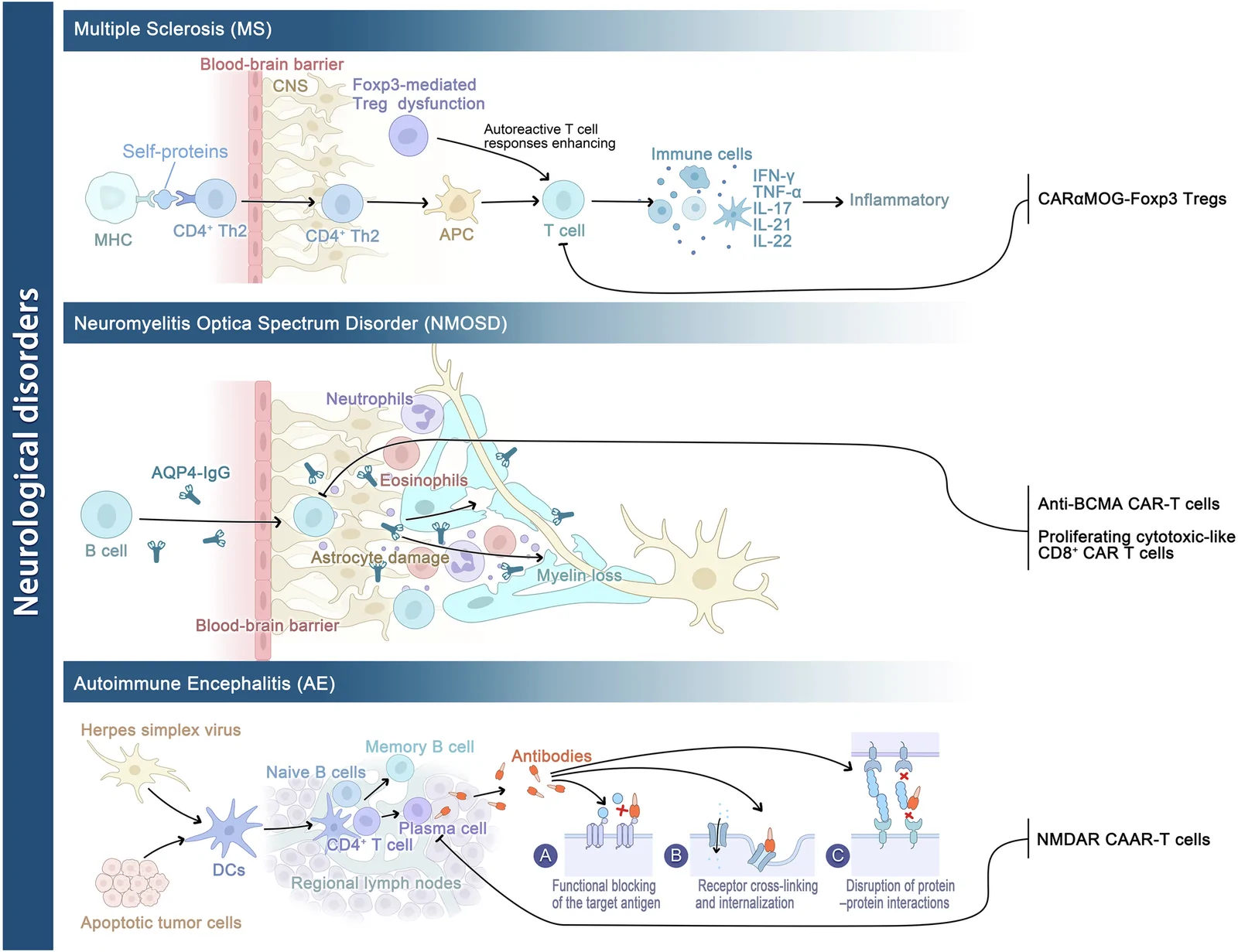
Mechanisms of neurological diseases. 1, Multiple sclerosis (MS) is triggered by self-antigens presented via MHC molecules that activate Th2 cells. These cells migrate to the central nervous system (CNS), where they are further activated by APCs, leading to the recruitment of additional immune cells and the initiation of an inflammatory response that damages neural tissue. CARαMOG-Foxp3 Tregs can suppress T cell activity, reduce cytokine levels, and alleviate inflammation. 2, Neuromyelitis optica spectrum disorder (NMOSD) is caused by plasmablasts that produce AQP4-IgG antibodies. These antibodies can cross the blood-brain barrier and mediate complement-dependent cellular damage within the CNS. Accumulation of neutrophils and eosinophils further contributes to the inflammatory response and demyelination. Anti-BCMA CAR-T cells and proliferating cytotoxic-like CD8⁺ CAR-T cells have been shown to effectively eliminate abnormally expanded plasmablasts and plasma cells in the cerebrospinal fluid. 3, Autoimmune encephalitis is triggered by antigens derived from virus-infected neurons or apoptotic tumor cells. This leads to a cascade that activates B cells, resulting in the production of autoantibodies. These antibodies (primarily targeting GABA, NMDAR, or LGI1) bind to neurons and cause neural injury. Currently, NMDAR-specific chimeric autoantibody receptor T cells (NMDAR CAAR-T cells) are being developed to selectively recognize and eliminate B cells and plasma cells that produce anti-NMDAR antibodies. GABA γ-aminobutyric acid, NMDAR N-methyl-D-aspartate-receptor, LGI1 leucine-rich gliom-nactivated-1

Despite these advances, significant challenges remain in Treg-based therapies for MS: Limited cell migration to target sites: Adoptively transferred Tregs often diminish in number during migration, with potential off-target accumulation in other organs. Delivery hurdles: While the olfactory pathway has been explored as a potential route for CNS delivery, systemic distribution via nasal vasculature remains a concern. Current tracking methods, such as olfactory nerve transmission monitoring, are limited to short-term observation (about 1 hour). And safety considerations: Exogenous Treg infusion may inadvertently suppress beneficial immune responses following CNS injury and even impair neuronal survival, necessitating caution in chronic neurodegenerative conditions.^300,301^ These findings underscore both the promise and complexities of CAR-Treg therapy for MS, emphasizing the need for refined delivery strategies and long-term safety assessments (Fig. 4).

CAR-Treg therapy represents a promising approach for multiple sclerosis, with MOG-specific CAR-Tregs demonstrating targeted migration to CNS regions and suppression of pro-inflammatory cytokines in EAE models. However, significant challenges require resolution before clinical translation. These include limited trafficking efficiency to intended sites with potential off-target organ accumulation, difficulties in reliable CNS delivery despite exploration of olfactory pathways, and safety concerns regarding potential suppression of beneficial immune responses and neuronal survival. Current tracking methods remain constrained to short-term observation. Future development must address these delivery limitations and conduct thorough long-term safety evaluations to realize the therapeutic potential of this approach.

##### Neuromyelitis optica spectrum disorder (NMOSD)

Neuromyelitis optica spectrum disorder (NMOSD) is a variant of MS characterized by severe, recurrent episodes and significant residual effects.^302^ Aquaporin-4-IgG antibodies (AQP4-IgG) are pathogenic in NMOSD, where plasma blasts producing AQP4-IgG and the antibodies themselves can breach the blood-brain barrier. This leads to complement-dependent cytotoxicity, immune cell chemotaxis, and various pathological outcomes. Recent clinical investigations of CT103A, a novel BCMA-targeting CAR-T therapy, have shown promising safety and efficacy profiles in AQP4-IgG-seropositive NMOSD patients.^303^

Study had revealed that abnormal expansion of B cell lineages, including plasma blasts, exist in both blood and cerebrospinal fluid (CSF) of NMOSD patients. Notably, approximately 13% of CSF B cells shared Ig-VH (immunoglobulin heavy chain variable region) sequences with those in blood samples, suggesting a central nervous system (CNS) origin. Single-cell transcriptomics and TCR/B-cell receptor (BCR) sequencing further characterized the clonal dynamics and transcriptional profiles of anti-BCMA CAR-T cells, highlighting the key role of proliferating cytotoxic-like CD8^+^ CAR-T cell clones in autoimmunity. These cells were derived mainly from circulating T cell subpopulations in the infusion product. Interestingly, CD8^+^ CAR-T cells showed increased transformation into NK-like T cells with early dysfunction rather than exhaustion. The study also demonstrated that CAR-T cells expressing CXCR3 and enriched for chemotactic gene programs could effectively cross the blood-CSF barrier and infiltrate CSF. These infiltrating CAR-T cells efficiently eliminated abnormally expanded plasmablasts and plasma cells in CSF, leading to reduced soluble BCMA levels and suppressed neuroinflammation. Moreover, the study observed that CD4^+^ CAR-T cells expressing CD44 and lower levels of L-selectin before infusion were associated with prolonged persistence, suggesting that an early memory phenotype may contribute to sustained CAR-T cell activity.^304^ Whether the cell can cross the blood-brain barrier, whether it can achieve effective concentration in the central nervous system, and whether its clearance of target cells is precise all require further investigation. Additionally, the extent of central nervous system damage caused by CRS (cytokine release syndrome) and how to monitor it remain unresolved issues.^305^ These findings highlight both the therapeutic potential and remaining knowledge gaps in applying CAR-T cell therapy for NMOSD, particularly regarding CNS-specific pharmacokinetics and safety profiling. The demonstrated ability of engineered T cells to traffic into CSF and modulate autoimmune B cell populations offers a promising foundation for further development, while underscoring the need for additional mechanistic and clinical investigations (Fig. 4).

##### Autoimmune encephalitis (AE)

Autoimmune encephalitis (AE) is a form of encephalitis driven by autoimmune mechanisms. Since the identification of anti-N-methyl-D-aspartate receptor (NMDAR) encephalitis in 2007, numerous autoantibodies targeting neuronal cell-surface or synaptic proteins have been discovered.^306^ AE accounts for approximately 10–20% of all encephalitis cases, with anti-NMDAR encephalitis being the most prevalent subtype, representing 54–80% of AE cases.^307^ Other notable forms include encephalitis associated with antibodies against leucine-rich glioma-inactivated protein 1 (LGI1) and γ-aminobutyric acid type B receptor (GABABR).^308–310^ Brain tissue biopsies from patients with NMDAR AE have revealed significant compartmentalized enrichment of CD20^+^ B cells and CD138^+^ plasma cells, suggesting that autoantibodies found in the cerebrospinal fluid (CSF) may be produced by activated immune cells infiltrating the central nervous system.^311–313^ Reincke et al. developed NMDAR-specific chimeric autoantibody receptor T cells (NMDAR CAAR-T), which consist of an NMDAR autoantigen fragment fused with a CD8 hinge and 41BB/CD3ζ intracellular signaling domains. In vitro, NMDAR CAAR-T cells were activated and expanded using K562 and Nalm6 cell lines co-expressing human monoclonal antibodies and luciferase (ffluc) as target cells. Upon activation, these CAAR-T cells secreted interferon-γ and granzyme B, leading to the selective lysis of target cells. The therapeutic efficacy of NMDAR CAAR-T cells was evaluated using ffluc as a reporter. In vivo, immunodeficient mice lacking natural killer cells and lymphocytes were co-injected with NMDAR antibodies and Nalm6 cells, followed by treatment with NMDAR CAAR-T cells. This resulted in a reduction of Nalm6 cells and elimination of the pathogenic antibodies, without any observed off-target toxicity or adverse effects.^314^ Current related experiments remain at the preclinical stage, and further investigation is required to determine their potential applicability. Moreover, there is a lack of experimental evidence regarding their extension to other antibody-based AE therapies (Fig. 4).

### Infectious diseases

**Fig. 5:**
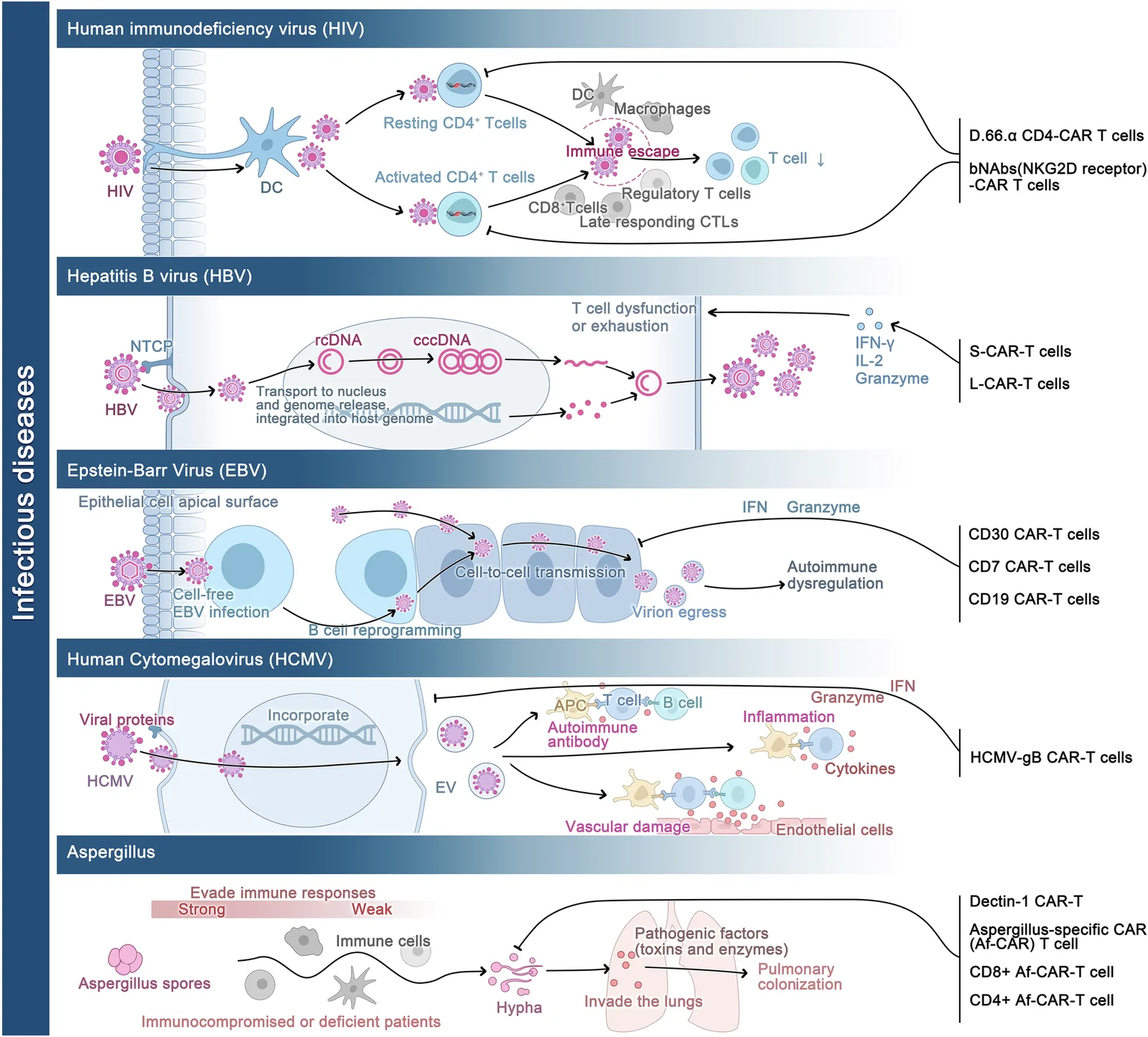
Mechanisms of infectious diseases. 1, After HIV infection, the virus is presented by dendritic cells (DCs) to CD4⁺ T cells, activating them while inserting fragments of viral genetic material into the host DNA. These infected cells become unrecognizable to the immune system and lose normal function, while continuing to produce and release new viruses. D.66.α CD4-CAR T cells and bNAbs (NKG2D receptor)-CAR-T cells can target and bind to HIV-infected CD4⁺ T cells. 2, Hepatitis B Virus (HBV) binds to NTCP receptors on the surface of hepatocytes, enters the cells via endocytosis, and uncoats to deliver its nucleic acids into the nucleus. There, the viral genome integrates into the host DNA, forming covalently closed circular DNA (cccDNA), which serves as a template for viral gene expression and replication. Newly assembled viruses are released to infect other hepatocytes. S-CAR-T cells and L-CAR-T cells can recognize and eliminate HBV-infected hepatocytes. 3, Epstein-Barr Virus (EBV) enters the body through the skin and infects B cells. Through cell-to-cell transmission, the virus spreads and reprograms B cells, resulting in immune dysregulation. CAR-T cells targeting CD30, CD7, and CD19 can identify and kill EBV-infected B cells. 4, Human Cytomegalovirus (HCMV) integrates viral proteins into extracellular vesicles of host cells. The virus causes damage to target organs by generating autoantibodies, recruiting immune cells, and injuring vascular endothelial cells. HCMV-gB CAR-T cells specifically recognize glycoprotein gB on the surface of HCMV and eliminate infected cells. In immunocompromised or immunodeficient patients, Aspergillus spores may evade immune recognition and develop into invasive hyphae that penetrate lung tissue, releasing toxins and enzymes that damage the lungs. Dectin-1 CAR-T cells recognize β-glucan on the surface of Aspergillus. Aspergillus-specific CAR (Af-CAR) T cells specifically target conserved antigens on Aspergillus, while CD8⁺ and CD4⁺ Af-CAR-T cells also recognize these conserved fungal antigens and help eliminate the infection. NTCP sodium taurocholate co-transporting polypeptide, CTLs cytotoxic T lymphocyte, rcDNA relaxed circular DNA, EV extracellular vesicles

Several persistent infectious agents employ sophisticated strategies to evade host immunity and establish chronic infections (Fig. 5). HIV primarily targets CD4^+^ T cells, integrating its genome into host DNA and escaping detection by dendritic cells, macrophages, and cytotoxic T cells, leading to progressive immune dysfunction.^315^ Similarly, HBV exploits the NTCP receptor on hepatocytes to deliver its genome, forming stable cccDNA that enables persistent viral replication and propagation.^316–318^ Herpesviruses demonstrate remarkable latency strategies: EBV maintains lifelong infection in B lymphocytes through episomal persistence and expression of EBNA proteins that promote immune evasion and oncogenesis,^319–324^ while HCMV incorporates viral proteins into extracellular vesicles to trigger autoimmunity and multi-organ damage.^325–327^ Unlike these viral pathogens, Aspergillus fungi invade immunocompromised hosts through airborne spores that germinate into invasive hyphae, overcoming respiratory defenses and releasing tissue-damaging toxins.^328,329^ Common to all these infections is their ability to subvert normal immune surveillance—whether through viral latency programs (HIV, HBV, EBV, HCMV) or structural adaptations (Aspergillus)—creating persistent reservoirs that challenge conventional therapies. This shared pathogenesis highlights the need for innovative approaches like CAR-T cells that can recognize and eliminate these evasive pathogens through engineered immune recognition.

Recent advances in CAR-T cell therapy have shown remarkable potential for treating persistent viral and fungal infections through innovative engineering approaches. For HIV infection, two primary strategies have emerged: CD4-based CAR-T cells utilizing truncated D1D2 domains to maintain HIV recognition while avoiding MHC class II interactions,^330–337^ and convertible CAR designs combining broadly neutralizing antibodies (bNAbs) with NKG2D receptors for selective targeting of infected cells.^330,331,338^ Structural optimizations, including LYC motif incorporation and hinge region modifications, have improved surface expression and antigen recognition.^339^ However, clinical translation faces challenges including variable viral reservoir targeting, CAR-T cell persistence, and tissue homing efficiency.^330,340–346^

In HBV, researchers have developed L-CAR-T and S-CAR-T cells targeting envelope proteins, demonstrating dual functionality through direct hepatocyte killing and IFN-γ/IL-2 secretion to combat cccDNA persistence.^347^ The S-CAR design shows particular promise with its conserved S-domain recognition, inducing rapid viral clearance in mouse models while exhibiting favorable liver tropism.^348^ Though only one clinical trial (NCT06251115) is currently evaluating HBV-specific CAR-T cells, highlighting the need to address viral escape and on-target/off-tumor effects. EBV-directed CAR-T therapies have primarily focused on malignancies, with CD19, CD30, and CD7-targeting constructs in clinical trials,^349,350^ while non-malignant EBV infections remain understudied. HCMV-specific CARs targeting glycoprotein complexes (particularly gB) demonstrate cytokine-mediated antiviral activity despite viral evasion mechanisms like UL37x1-mediated inhibition of cytotoxicity.^351–353^ Costimulatory domain incorporation (e.g., 4-1BB) has enhanced functionality^354–357^ though GVHD risks require careful evaluation.

For invasive aspergillosis, CAR-T strategies have evolved from broad-spectrum Dectin-1 targeting^50^ to more specific AB90-E8-directed designs that show superior fungal clearance through perforin/granzyme release and macrophage activation.^358^ However, unidentified antigen identity and manufacturing delays for acute infections remain substantial barriers.

While these CAR-T approaches demonstrate impressive preclinical results across HIV,^359^ HBV,^347^ EBV,^360^ HCMV,^48^ and Aspergillus^50^ infections, several shared limitations must be addressed: antigenic variability and escape, tissue-specific trafficking and persistence, manufacturing complexities for acute infections, safety concerns including cytokine release and on-target/off-tumor effects, limited clinical trial activity, and some trials have only shown effectiveness in animal models. The field now requires optimized CAR designs combining multiple effector functions, improved delivery strategies for viral reservoirs, and accelerated manufacturing platforms to realize the full therapeutic potential against these challenging infections.

CAR-T cell therapy demonstrates significant potential against persistent infections through pathogen-specific engineering, including CD4-based CARs for HIV, envelope-targeting constructs for HBV, glycoprotein-directed approaches for herpesviruses, and Dectin-1/AB90-E8 CARs for Aspergillus. However, these developments face substantial challenges: antigenic variability, limited tissue homing to reservoir sites, manufacturing complexities for acute infections, and safety concerns including on-target/off-tumor effects. Current clinical evaluation remains limited, with only one active HBV CAR-T trial (NCT06251115) identified. Future progress requires optimized CAR designs integrating multiple effector functions, enhanced delivery strategies to viral reservoirs, and improved manufacturing platforms to address these persistent infections effectively.

### Aging and senescence

#### Cellular senescence-associated diseases

Cellular aging emerges from sustained cell cycle arrest triggered by diverse stressors including replicative stress, oxidative damage, mitochondrial dysfunction, radiation exposure, chemotherapy, and traumatic injury. These insults activate two primary pathways: (1) the INK4/ARF locus, where ARF inhibits MDM2 to stabilize p53, while p16^INK^4a blocks CDK4/6-mediated Rb phosphorylation, maintaining E2F binding and G1 arrest; and (2) DNA damage responses, where ATM/ATR kinases activate p53 to induce p21, subsequently inhibiting Cdk2-mediated pRb phosphorylation and similarly enforcing G1 arrest.^361^ Senescent cells subsequently develop the senescence-associated secretory phenotype (SASP), releasing cytokines, chemokines, and matrix metalloproteinases that drive pathology through multiple mechanisms: immune cell recruitment (macrophages, dendritic cells, neutrophils), chronic inflammation, senescence reinforcement, paracrine senescence induction, and cancer progression.^362^ Age-related accumulation of senescent cells results from progressive tissue damage and declining immune surveillance, contributing to organismal aging.^363,364^ Genetic ablation studies demonstrate that selective removal of senescent cells can ameliorate age-related metabolic dysfunction and physical decline.^127,365–367^ While senolytic drugs partially replicate these benefits, their mechanisms often remain unclear and require continuous administration.^36,368^

**Fig. 6:**
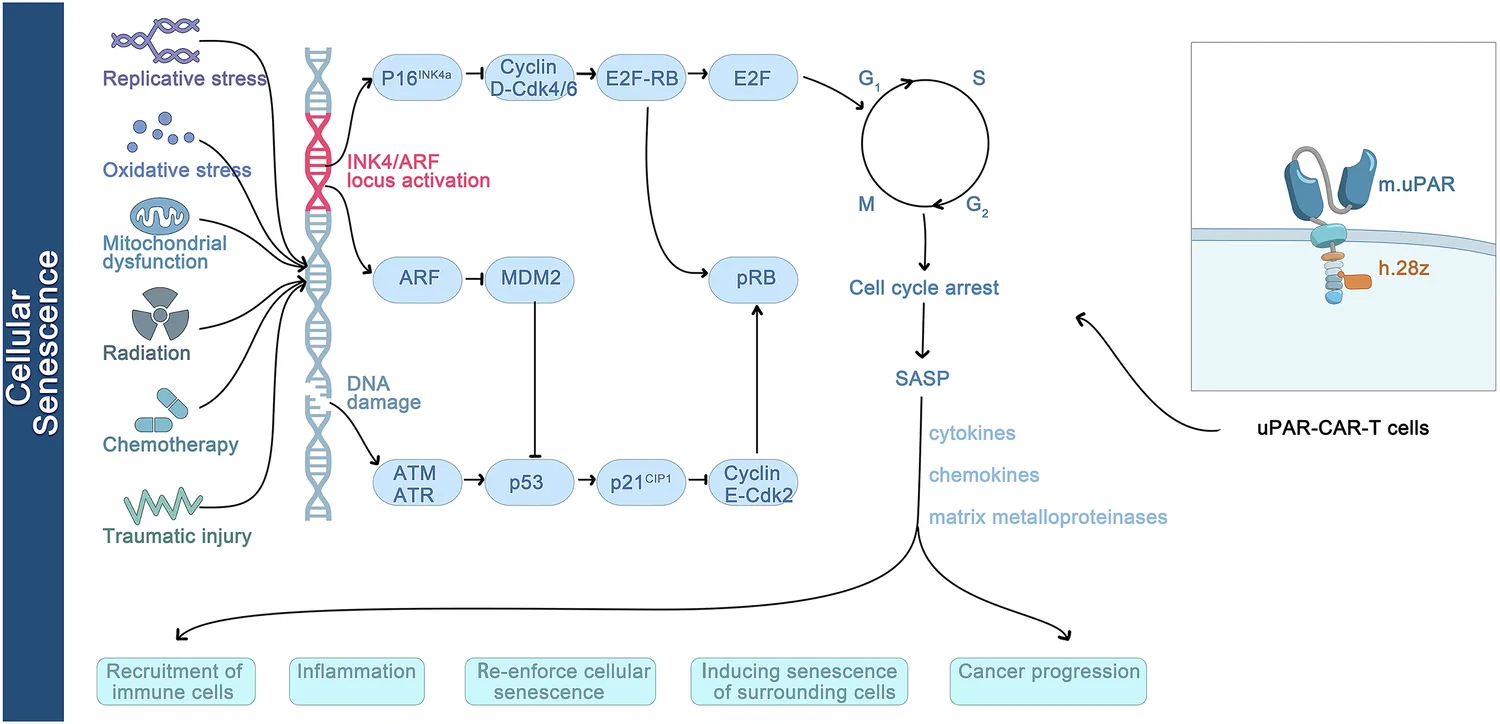
Mechanisms of aging and senescence. Various damaging factors can lead to DNA damage or activation of the INK4/ARF gene, resulting in the upregulation of downstream effectors such as p16^INK4a^, p53, and p21. This process weakens the inhibitory effect of MDM2, leading to increased binding between RB and E2F, ultimately causing cell cycle arrest. Arrested cells develop a senescence-associated secretory phenotype (SASP), which includes cytokines, chemokines, and matrix metalloproteinases. These factors contribute to pathogenesis by recruiting immune cells, triggering inflammation, reinforcing senescence in an autocrine and paracrine manner, and potentially promoting cancer progression. uPAR CAR-T cells, which combine a human intracellular co-stimulatory domain with a murine extracellular antigen-recognition receptor, can specifically recognize and eliminate senescent cells

Recent advances identify urokinase-type plasminogen activator receptor (uPAR) as a promising target, with uPAR-positive cells increasing proportionally with age.^36^ Engineered uPAR CAR-T cells (m.uPAR-h.28z) incorporating anti-muPAR scFv with hCD28/CD3ζ signaling domains demonstrate remarkable properties^368^: specific elimination of senescent cells in juvenile and aged mice without tissue toxicity, improved glucose homeostasis and metabolic function in both physiological aging and high-fat diet models, long-term persistence (> 15 months) following single low-dose administration, prevention of age- and diet-induced metabolic decline when administered prophylactically, enhanced pancreatic β-cell function and insulin sensitivity in metabolic syndrome models, and reduced senescent cell burden across multiple tissues (pancreas, liver, adipose). These findings corroborate earlier work using GPNMB vaccination against senescent cells.^369^ Notably, uPAR CAR-T cells show particularly strong activity in aged mouse livers and spleens, with protective effects lasting over half the murine lifespan.^368^ The durability of response after single-dose administration contrasts favorably with conventional senolytics. The field faces significant challenges in target identification due to senescent cell heterogeneity, making universal surface antigens unlikely.^370,371^ However, this diversity may enable precision targeting of specific senescent subpopulations. Alternative targets under investigation include NKG2D ligands (MICA, MICB, ULBP1-5), though their broad expression patterns present manufacturing and toxicity control challenges.^372–374^ These developments highlight the potential of immunotherapeutic approaches to address fundamental aging mechanisms while underscoring the need for continued target discovery and validation (Fig. 6).

uPAR-targeting CAR-T cells represent a significant advancement in senolytic therapy, demonstrating specific elimination of senescent cells across multiple tissues with durable effects from single-dose administration. These cells improve metabolic function and extend healthspan in aging models, outperforming conventional senolytics through their persistent activity. However, the field faces the fundamental challenge of senescent cell heterogeneity, which complicates universal target identification. While uPAR shows promise, alternative targets like NKG2D ligands present manufacturing and toxicity concerns. Future progress depends on discovering additional surface markers specific to senescent subpopulations and validating their therapeutic potential, balancing precision with practicality in clinical translation.

### Cardiovascular and metabolic disorders

#### Cardiac fibrosis

Cardiac fibrosis, a key pathological feature of heart failure, develops through two distinct patterns: reparative fibrosis forming organized scar tissue post-MI to stabilize necrotic areas, and reactive fibrosis characterized by interstitial ECM deposition in non-ischemic cardiomyopathy or surviving myocardium, disrupting tissue architecture.^375–380^ The process is driven by mast cell-derived mediators activating fibroblasts, leading to excessive ECM accumulation that impairs contractility and promotes arrhythmias.^378–385^ While initially adaptive (e.g., post-MI scar stabilization), fibrosis becomes maladaptive, though evidence suggests reversibility particularly in HFpEF.^386,387^ Therapeutic strategies focus on targeting ECM degradation, myofibroblast elimination, and fibroblast activation pathways.^28,388–390^

**Fig. 7:**
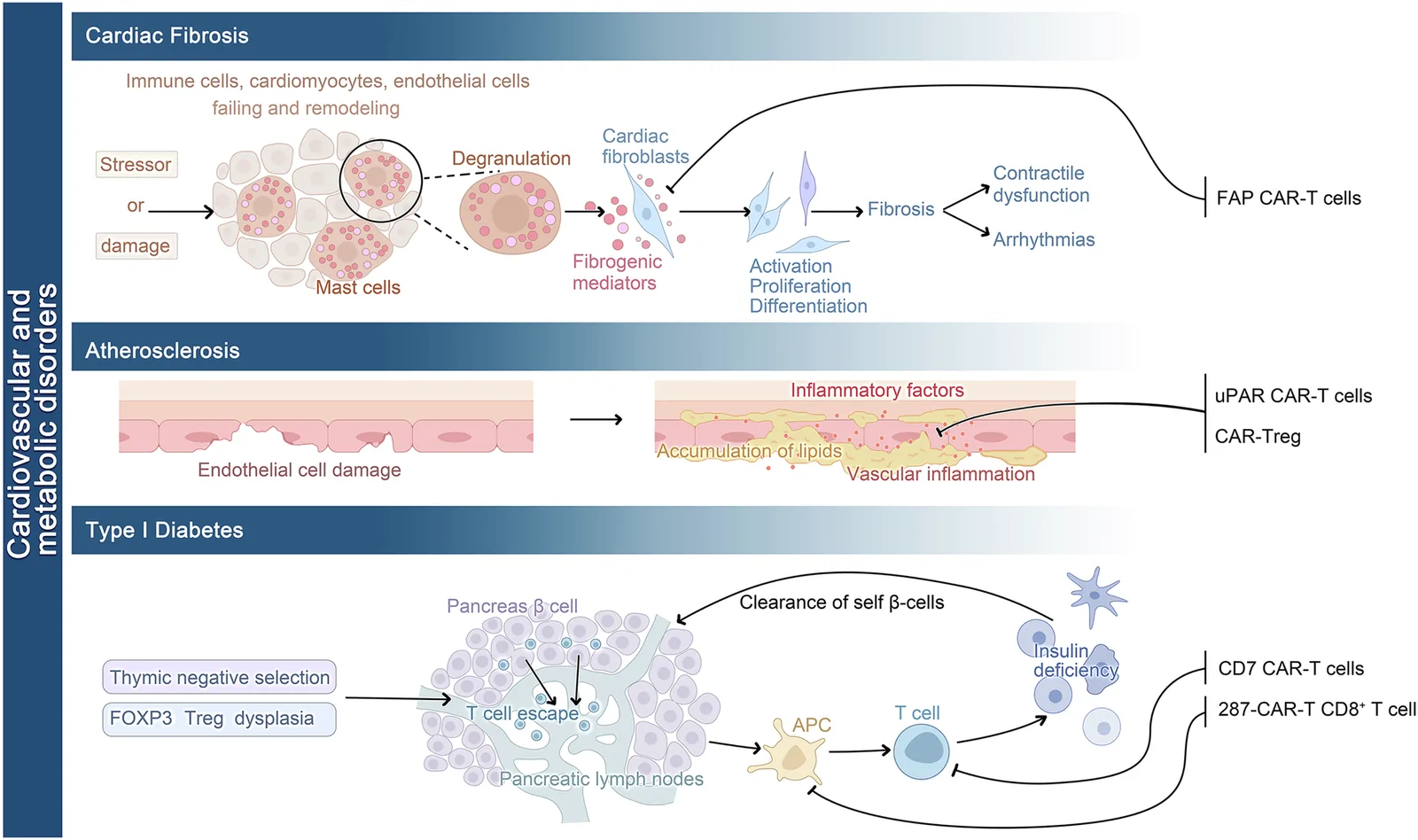
Mechanisms of cardiovascular and metabolic disorders. 1, Cardiac fibrosis occurs following tissue injury, during which the number of mast cells increases. These cells undergo degranulation and release various fibrotic mediators that activate, differentiate, and promote the proliferation of fibroblasts. This leads to excessive local collagen deposition, resulting in impaired contractile function or arrhythmia. FAP-CAR-T cells can recognize fibroblast surface antigens and exert a fibroblast-clearing effect. 2, Atherosclerosis is characterized by chronic inflammation and localized autoimmune responses that cause lipid accumulation in blood vessels. These deposits further trigger recurring vascular inflammation. uPAR-CAR-T cells can recognize and eliminate damaged vascular endothelial cells, preventing downstream inflammatory reactions. CAR-Tregs can suppress local immune responses, thereby reducing inflammation and autoimmunity in affected vascular regions. 3, Type 1 diabetes is caused by defective development of FOXP3⁺ Tregs, leading to impaired immune tolerance. As a result, pancreatic β-cells are targeted and destroyed by the immune system, resulting in insulin deficiency. The lack of immune suppression allows infection or stress-related inflammatory factors to further activate T and B cells, amplifying the autoimmune attack on β-cells. Universal CD7 CAR-T cells and 287 CAR CD8⁺ T cells can target and eliminate autoreactive immune cells, thereby reducing the autoimmune response

In mouse models of hypertensive heart injury and fibrosis, researchers designed CAR-T cells targeting fibroblast activation protein (FAP) to specifically eliminate myofibroblasts. Adoptive transfer of FAP CAR-T cells significantly reduced cardiac fibrosis and restored cardiac function, with effects observed exclusively in the heart.^28,84,391–396^ These findings build upon existing angiotensin pathway inhibitors (ACEi/ARBs) that indirectly modulate fibroblast activity,^388^ while offering potential advantages through direct, targeted myofibroblast elimination. The success of these approaches hinges on the dynamic nature of cardiac fibrosis and the abundance of targetable myofibroblasts in fibrotic hearts,^28^ which first be identified as therapeutic targets in foundational preclinical work (Fig. 7).

#### Atherosclerosis

Atherosclerosis develops through a complex interplay of chronic inflammation and autoimmune responses. The pathogenic cascade initiates with intimal injury, triggering recruitment of immune cells (monocytes/macrophages, neutrophils, and lymphocytes) into the vascular wall. This infiltration promotes lipid accumulation and sustains vascular inflammation, driving disease progression.^397^ While most recruited immune cells exacerbate atherosclerosis, CD4^+^ regulatory T cells (Tregs) serve a protective function by maintaining immune homeostasis through suppression of pro-inflammatory responses. Despite their relatively low abundance within atherosclerotic plaques, Tregs play a crucial role in preserving immune tolerance.^398–400^

There are currently two proposed solutions. One is uPAR CAR-T, where endothelial cell-induced chronic inflammation itself triggers a series of inflammatory responses due to subsequent degenerative changes. By eliminating the abnormally displayed endothelial cells, it can reduce the immune response triggered.^401^ The other is regulatory CD4^+^ Tregs. Engineered Tregs: These counteract hyperlipidemia-induced endothelial damage by suppressing autoimmune responses and preserving vascular endothelial function.^402^ However, significant challenges remain in translating these approaches. Infusion of polyclonally expanded Treg cells with unknown antigen specificity cannot effectively suppress target cells and inhibit undesirable immune responses and may lead to unwanted side effects such as systemic immunosuppression and reactivation of latent infections. Therefore, the use of polyclonal infusions in clinical trials is often the main reason for the low efficacy of the adopted therapies. CAR Treg cell infusion may induce so-called cytokine storm and neurotoxicity.^402^ Furthermore, the field faces ongoing difficulties in target identification and engineering approaches suitable for precise immune modulation.^402^ These limitations highlight the need for continued refinement of cellular therapies to achieve selective immunomodulation without compromising protective immune functions in atherosclerosis (Fig. 7).

#### Type 1 diabetes

Type 1 diabetes mellitus (T1D) develops through autoimmune destruction of pancreatic β-cells, primarily driven by excessive activation of effector T cells within pancreatic islets.^11^ This process involves multiple pathological mechanisms: (1) deficient regulatory T cell (Treg) activity fails to control autoreactive T cell expansion against islet autoantigens (insulin, ZnT8, GAD65); (2) impaired thymic selection or defective FOXP3^+^ Treg development leads to loss of self-tolerance; and (3) β-cell-specific T cells migrate to pancreatic lymph nodes where they encounter antigen-presenting cells (APCs) displaying pancreatic antigens, subsequently differentiating into inflammatory effector T cells that infiltrate islets and mediate β-cell destruction.^403–406^ Current treatments involve CD7 CAR-T (universal CAR-T), with at least one positive islet autoantibody (e.g., GADA, IA-2A, IAA, ZnT8). In 2021, a study demonstrated that CAR-T could restore normal insulin secretion in mice. In 2022, CAR-Tregs were found to reverse Type 1 diabetes. Major histocompatibility complex (MHC) II gene polymorphisms, especially HLA-DQ, are crucial in Type 1 diabetes development.^407,408^ The disease’s strong genetic component involves MHC II polymorphisms, particularly HLA-DQ variants. In non-obese diabetic (NOD) mice, the insulin B chain peptide 9-23 serves as a critical autoantigen when presented by MHCII (I-A^g7^), activating pathogenic T cells.^409^ A proof-of-concept study engineered CAR-T cells targeting I-A ^g7^-B:9-23, evaluating the cytotoxicity of CAR-engineered CD8^+^ T cells against APCs presenting I-A ^g7^-B:9-23. Results showed 287-CAR-T CD8^+^ T cells were specific in detecting and killing antigen-presenting cells (APCs) presenting I-A ^g7^-B:9-23, with higher proliferation and homing rates in pancreatic lymph nodes compared to spleens and inguinal lymph nodes.^47^ Although a single infusion of CAR-T CD8^+^ cells did not achieve complete cure, disease onset was delayed, indicating reversible protection.^47^

In these studies, allogeneic infusion of CAR-T cells can induce GvHD, especially chronic GvHD that persists over the long term. Despite these advances, significant challenges remain. Further research is needed in the design of CAR-T therapies.^410^ Additionally, the issue of Treg persistence after in vivo infusion also needs to be addressed.^411^ Moreover, the balance between effective autoimmunity suppression and general immune competence requires precise modulation. These findings highlight both the promise and complexities of cellular therapies for T1D, where successful translation will require solutions to persistence, specificity, and safety challenges while leveraging the unique capacity of engineered immune cells to target autoimmune processes at their source (Fig. 7).

CAR-T therapies demonstrate promising applications across cardiovascular and metabolic diseases. FAP-targeted CAR-T cells effectively reduce cardiac fibrosis in murine models, while uPAR CAR-T and engineered Tregs offer potential approaches for atherosclerosis. In type 1 diabetes, CAR-T cells targeting specific MHC-peptide complexes show capability to delay autoimmune destruction. However, these approaches face significant challenges including target identification precision, potential for graft-versus-host disease, limited persistence of therapeutic cells, and the critical balance between effective autoimmunity suppression and preserved immune function. The field requires continued refinement of CAR designs and thorough investigation of long-term safety profiles before clinical translation can be realized.

## Clinical translation and trials

The latest clinical trial results and registered clinically relevant experiments and advancements.

Although no clinical drugs for non-tumor diseases have been approved yet, related clinical trials are underway, most of which are in Phase I or pre-Phase I stages (Table 1). Several of these experiments have yielded significant breakthroughs, for example, the allogeneic universal CD19 CAR-T cell therapy developed by Xu’s team was administered to three patients with severe autoimmune diseases.^412^ Specifically, an SRP-IMNM patient who received TyU19 cell therapy did not experience any fever or cytokine storm symptoms. Additionally, the patient’s Total Improvement Score (TIS) rapidly increased from a baseline of 72.5 to 100 within two months and remained stable during follow-up. Imaging and pathological examinations also showed a significant reduction in muscle inflammation. Two diffuse cutaneous systemic sclerosis (dcSSc) patients who received TyU19 cell therapy showed a significant increase in CRISS scores within a few months. Regarding skin fibrosis, the modified Rodnan skin scores (mRSS) of both patients decreased significantly.^413^ Similarly, Yang et al. evaluated the safety and efficacy of allogeneic CD19-targeted CAR-T cells (TyU19) in refractory SLE. The study enrolled four young female patients (aged 22–24) with treatment-refractory lupus. All four patients demonstrated sustained clinical improvement in signs and symptoms. At the 3-month follow-up, all met the Systemic Lupus International Collaborating Clinics (SLICC) criteria for sustained remission (SRI-4). Notably, peripheral BCMA^+^ and CD19−BCMA^+^ plasma cells declined in all patients, suggesting that TyU19 infusion may facilitate plasma cell reconstitution. Consistent with observations in autologous CAR-T therapies, most reconstituted B cells were naive B cells, with marked reductions in memory B cells and plasma cells. One patient who discontinued post-intervention immunosuppressants maintained durable drug-free remission, highlighting the potential advantages of allogeneic CAR-T cells as a promising therapy for refractory SLE (NCT05988216).^414^

**Table 1:** Ongoing clinical trials for non-oncological diseases

Disease Type	Diseases	Target	Drug name	Phases	Stage	Efficacy/safety outcomes	Trail number
Autoimmune diseases	Lupus nephritis	B cells	ATA3219	PHASE1	Not yet recruiting	Incidence and severity of treatment-emergent adverse events	NCT06429800
Autoimmune diseases	Chronic graft versus host disease (cGVHD)	T cells	CD6-CAR Tregs	PHASE1	Recruiting	Change chronic graft versus host disease (cGVHD) severity.	NCT05993611
Autoimmune diseases	SLE	B cells	BH002 injection (CD19-BCMA CAR-T cells)	PHASE1 | PHASE2	Not yet recruiting	Incidence and severity of dose limiting toxicities (DLTs), CD19/BCMA chimeric antigen receptor (CAR) T cells related specifications outlined	NCT06350110
Autoimmune diseases	SLE, Anca-associated Vasculitis, and Idiopathic Inflammatory Myopathy(IIM)	B cells	IMPT-514 CART Cell	EARLY_PHASE1	Not yet recruiting	DLTs, serious adverse events (SAE) and treatment-emergent adverse events (TEAEs).	NCT06462144
Autoimmune diseases	Autoimmune disease	B cells	T cell injection targeting CD19 chimeric antigen receptor UTAA09	NA	Recruiting	Type, frequency, and severity of adverse events (AEs) and laboratory abnormalities	NCT06361745
Autoimmune diseases	IIM	B cells	CABA-201	PHASE1 | PHASE2	Recruiting	Incidence and severity of AEs	NCT06154252
Autoimmune diseases	Autoimmune disease	B cells	KYV-101	PHASE1	Not yet recruiting	Incidence and severity of AEs	NCT06152172
Autoimmune diseases	Autoimmune disease	B cells	CC-97540	PHASE1	Recruiting	Number of AEs. Proportion remission in SLE (DORIS) remission.	NCT05869955
Autoimmune diseases	Autoimmune disease	B cells	T cell injection targeting CD19 chimeric antigen receptor	EARLY_PHASE1	Not yet recruiting	Type, frequency, and severity of AEs and laboratory abnormalities.	NCT06417398
Autoimmune diseases	Crohn Disease, Ulcerative Colitis	T cells	CD7 CAR T-cells	EARLY_PHASE1	Recruiting	Incidence of TEAEs and AEs. Overall response rate (ORR).	NCT05239702
Autoimmune diseases	SLE	B cells	BAFF CAR-T Cells (LMY-920)	PHASE1	Not yet recruiting	Safety of the Treatment, The rate of AEs, Systemic Lupus Erythematosus Disease Activity Index (SELENA-SLEDAI)	NCT06340750
Autoimmune diseases	SLE	B cells	Assigned Interventions CD19/BCMA CAR T-cells	EARLY_PHASE1	Unknown	DLTs. Autoantibody detection. Detect the lupus erythematosus antibody titer in vivo	NCT05030779
Autoimmune diseases	SLE	B cells	BCMA/CD19 CAR-T cells	PHASE1 | PHASE2	Recruiting	DLTs. Rate of successful manufacture and expansion of the CD19/BCMA CAR T cells.	NCT06428188
Autoimmune diseases	SLE	B cells	Anti-CD19-CAR-T cells	PHASE1	Unknown	AEs. Number of patients with tumor response. Detection of transferred T cells in the circulation using quantitative -PCR	NCT03030976
Autoimmune diseases	SLE	B cells	CAR T-cell therapy	PHASE1	Recruiting	Disease activity of SLE, The Safety of Estrogens in Lupus Erythematosus National Assessment (SELENA) - Systemic Lupus Erythematosus Disease Activity Index (SLEDAI); Complete response rate of lupus nephritis	NCT06150651
Autoimmune diseases	SLE	B cells	Anti-CD19 CAR T cell therapy	PHASE1 | PHASE2	Recruiting	Cytokine Release Syndrome (CRS) and of CAR T cell-associated neurotoxicity (ICANS). ORR	NCT06347718
Autoimmune diseases	SLE	B cells	Anti-CD19-CAR-T cells	PHASE1	Recruiting	Incidence and severity of AEs and SAEs, The efficiency of CAR-T cell in refractory childhood-onset SLE. Cellular kinetics. Autoantibody detection	NCT06222853
Autoimmune diseases	SLE	B cells	Obecabtagene autoleucel (obe-cel)	PHASE1	Recruiting	Incidence and severity of AEs and SAEs, The efficiency of CAR-T cell in refractory childhood-onset SLE. Cellular kinetics, Autoantibody detection	NCT06333483
Autoimmune diseases	SLE	B cells	RJMty19 (CD19-CAR-DNT cells)	PHASE1	Not yet recruiting	DLT and remission rate	NCT06340490
Autoimmune diseases	SLE	B cells	SCRI-CAR19v3	PHASE1	Not yet recruiting	Incidence of TEAEs. Safety and Tolerability	NCT06465147
Autoimmune diseases	SLE	B cells	BRL-301	NA	Recruiting	DLT and remission rate	NCT05988216
Autoimmune diseases	SLE	B cells	ATHENA CAR-T	PHASE1	Not yet recruiting	DLT and remission rate	NCT06373991
Autoimmune diseases	Immune Nephritis, Lupus Nephritis	B cells	Assigned Interventions CD19/BCMA CAR T-cells	EARLY_PHASE1	Recruiting	DLT and Concentration of CAR-T cells	NCT05085418
Autoimmune diseases	SLE, Lupus Nephritis	B cells	CD19- BCMA CAR-T cells	PHASE1 | PHASE2	Not yet recruiting	DLT and Rate of successful manufacture and expansion of the CD19/BCMA CAR T cells	NCT06350110
Autoimmune diseases	Lupus Nephritis	B cells	KYV-101 anti-CD19 CAR-T cell therapy	PHASE1 | PHASE2	Recruiting	DLT and levels of KYV-101 CAR-positive T cells in the blood	NCT06342960
Autoimmune diseases	Lupus Nephritis, SLE	B cells	ATA3219	PHASE1	Not yet recruiting	Maximum Observed Plasma Concentration (Cmax) of ATA3219 and (Hybrid SELENA-SLEDAI) Index and DLT	NCT06429800
Autoimmune diseases	Lupus Nephritis	B cells	KYV-101 anti-CD19 CAR-T cell therapy	PHASE1 | PHASE2	Recruiting	Cmax and (Hybrid SELENA-SLEDAI) Index and DLT	NCT05938725
Autoimmune diseases	SLE, Lupus Nephritis	B cells	IMPT-514	PHASE1 | PHASE2	Recruiting	DLT and proportion of achieving DORIS remission	NCT06153095
Autoimmune diseases	SLE	B cells	Anti-CD19-CAR-T cells	EARLY_PHASE1	Not yet recruiting	DLT and percentage of anti-CD19 -CAR-T cells can be successfully manufactured	NCT06420154
Autoimmune diseases	SLE	B cells	Anti-CD19-CD3E-CAR-T cells	NA	Recruiting	DLT and remission rate	NCT06373081
Autoimmune diseases	SLE	B cells	Single dose of CNCT19	EARLY_PHASE1	Recruiting	DLT and overall survival (OS)	NCT06316791
Autoimmune diseases	Relapsed/Refractory, SLE	B cells	BCMA-CD19 cCAR T cells	PHASE1	Recruiting	Autoantibody detection and the number and incidence of adverse events after BCMA-CD19 cCAR T cell infusion	NCT05474885
Autoimmune diseases	Lupus Nephritis, ANCA Associated Vasculitis	B cells	PRG-1801; AR-T against BCMA	NA	Recruiting	DLT and Remission rate	NCT06277427
Autoimmune diseases	SLE	B cells	BRL-301	NA	Recruiting	DLT and PK parameters	NCT05859997
Autoimmune diseases	Lupus Nephritis	B cells	PRG-1801	EARLY_PHASE1	Recruiting	DLT and Remission rate	NCT06497387
Autoimmune diseases	Lupus Nephritis	B cells	PRG-2311	EARLY_PHASE1	Recruiting	DLT and Remission rate	NCT06497361
Autoimmune diseases	SLE	B cells	MB-CART19.1	PHASE1 | PHASE2	Not yet recruiting	DLT and Levels of immunology parameters	NCT06189157
Autoimmune diseases	Lupus Erythematosus, SLE	B cells	SC291	PHASE1	Recruiting	DLT and Remission rate	NCT06294236
Autoimmune diseases	SLE	B cells	CD20/BCMA-directed CAR-T cells	PHASE1	Recruiting	DLT and Time to response (TTR), Serum cytokines, Detection of B cells concentration changes in bone marrow, muscle or kidney by flow cytometry	NCT06249438
Autoimmune diseases	SLE, ANCA Associated Vasculitis, IIM	B cells	IMPT-514 CART Cell Injection	EARLY_PHASE1	Enrolling by invitation	DLT	NCT06462144
Autoimmune diseases	SLE, Lupus Nephritis	B cells	YTB323	PHASE1 | PHASE2	Recruiting	DLT and CAR transgene levels; Definitions of remission in Systemic Lupus Erythematosus (DORIS) criteria	NCT05798117
Autoimmune diseases	SLE	B cells	T cell injection targeting CD19 chimeric antigen receptor	NA	Recruiting	DLT and Cmax, The maximum amplified concentration (Cmax); Concentration levels of CAR-T-associated serum cytokines	NCT06361745
Autoimmune diseases	SLE, Lupus Nephritis	B cells	CABA-201	PHASE1 | PHASE2	Recruiting	DLT and evaluate disease related biomarkers	NCT06121297
Autoimmune diseases	SLE	B cells	Relma-cel	PHASE1	Not yet recruiting	DLT and SELENA-SLEDAI,	NCT06297408
Autoimmune diseases	Lupus Nephritis	B cells	ADI-001	PHASE1	Not yet recruiting	DLT	NCT06375993
Autoimmune diseases	Refractory SLE	B cells	CNCT19 cell	EARLY_PHASE1	Enrolling by invitation	DLT	NCT05930314
Autoimmune diseases	SLE, Systemic Sclerosis	B cells	CC-97540	PHASE1	Recruiting	DLT and remission rate	NCT05869955
Autoimmune diseases	SLE	B cells	T cell injection targeting CD19 chimeric antigen receptor	EARLY_PHASE1	Not yet recruiting	DLT and Peak Plasma Concentration; content of CD19 positive cells in peripheral blood	NCT06417398
Autoimmune diseases	Lupus Nephritis, ANCA-associated Vasculitis	B cells	FKC288	PHASE1	Recruiting	The proportion of AEs. The proportion of participants who achieve partial remission (PR) or complete remission (CR) of renal. OS.	NCT06285279
Autoimmune diseases	IIM	B cells	CABA-201	PHASE1 | PHASE2	Recruiting	Incidence and severity of AEs	NCT06154252
Autoimmune diseases	Autoimmune Diseases	B cells	Anti-BCMA and CD19 CART cells	PHASE1	Recruiting	DLT and remission rate	NCT06503224
Autoimmune diseases	Systemic Sclerosis (SSc)	B cells	Anti-CD19 CAR-T cells	PHASE1	Recruiting	Change in the modified Rodnan Skin Score (mRSS) from baseline	NCT06792344
Autoimmune diseases	Systemic Sclerosis (SSc)	B cells	UTAA91	EARLY_PHASE1	Not yet recruiting	Incidence and severity of AEs	NCT07000682
Autoimmune diseases	Systemic Sclerosis (SSc)	B cells	CABA-201	PHASE1 | PHASE2	Recruiting	Incidence and severity of AEs	NCT06328777
Infectious diseases	EBV	Specific protein of EBV	BIOSG-BRG-01	PHASE1	Completed	The maximum CAR-T dose. The incidence AEs, SAEs, adverse events of special interest (AESI), CRS, ICANS and TEAEs.	NCT05864924
Infectious diseases	EBV	EBV infected cells	EBV-TCR-T (YT-E001) cells	PHASE2	Unknown	Safety and feasibility	NCT03648697
Infectious diseases	HBV	HBV	QY-1-T	Not Available	Recruiting	Tolerability of QY-1-T and determine the maximum tolerated dose (MTD), antitumor activity of QY-1-T in subjects with HBV-related HCC, changes in serum levels of HBsAg and serum levels of HBV-DNA before and after QY-1-T infusion	NCT06251115
Infectious diseases	HIV	HIV-infected cells	VC-CAR-T	PHASE1	Recruiting	Incidence of TEAEs, the HIV loads in the peripheral blood Mono-nuclear cells and plasma, the remaining concentration of VC-CAR-T cells in patients, the number of HIV-specific CD4, CD8 and their activity after receiving CAR-T cell therapy.	NCT03240328
Infectious diseases	HIV	HIV-infected cells	CMV-specific HIV-CAR T Cells	EARLY_PHASE1	Recruiting	DLT, post CAR T cell infusion, CD4 + T cell count and HIV RNA levels, percentage and persistence of EGFR + CD3 + T cells, number of CMV/HIV-CAR T cells, cytokine levels in the peripheral blood.	NCT06252402
Infectious diseases	HIV	HIV-infected cells	CAR-T cells	PHASE1	Unknown	Evaluation the safety and AEs of CAR-T-cell treatment, evaluate the change of HIV-1 latent reservoir in peripheral blood. Measure the cellular kinetics of CAR-T cells after infusion by RT-PCR and flow cytometry.	NCT04863066
Infectious diseases	HIV	HIV-infected cells	Chidamide with CAR-T or TCR-T cell therapy	PHASE1	Completed	Incidence of treatment-associated AEs, the HIV loads in the peripheral blood Mono-nuclear cells and plasma, the number of HIV-specific CD4,CD8,VC-CAR-T and TCR-T cells after receiving the therapy.	NCT03980691
Infectious diseases	HIV	HIV-infected cells	LVgp120duoCAR-T cells	PHASE1| PHASE2	Recruiting	Number of AEs, persistence of LVgp120duoCAR-T cells in blood during therapy.	NCT04648046
Infectious diseases	HIV	HIV-infected cells	CD4 CAR + CCR5 ZFN T-cells	PHASE1	Active not recruiting	Number of TEAEs. The percentage of enriched modified CD4 CAR + CCR5 ZFN cells and their subsets. Compare viral set point HIV RNA level. Size of latent HIV reservoir.	NCT03617198
Infectious diseases	HIV	HIV-infected cells	KD-247	PHASE1	Completed	Change in CD4 count, HIV viral load, and absolute lymphocyte count.	NCT05784415
Neurological disorders	Autoimmune encephalitis	B-cell maturation antigen (BCMA) is expressed on the surface of plasma cells	CT103A	EARLY_PHASE1	Recruiting	Types and incidence of DLT, changes of concentration of soluble BCMA in the peripheral blood after CT103A infusion, and clinical symptom relief.	NCT04561557

CD19 CAR-T therapy has shown promise in autoimmune diseases, particularly systemic lupus erythematosus (SLE). Some case reports have indicated that patients experienced symptom relief without significant adverse reactions. This includes remission of SLE according to DORIS criteria and improvements in laboratory parameters, such as seroconversion of anti-double-stranded DNA antibodies.^114^ Additionally, low C3 and C4 levels normalized, and the Systemic Lupus Erythematosus Disease Activity Index score (with the SELENA [Safety of Estrogens in Lupus National Assessment] modification) decreased from 16 at baseline to 0 at follow-up.^150^

## Challenges and risk-benefit analysis of CAR-T cell therapy in non-oncological diseases

### Safety

Cytokine release syndrome (CRS): After CAR-T cells are infused into the body, they recognize and activate against the body’s tumor cells, releasing large amounts of cytokines. This further activates a large number of immune cells (T cells, B cells, macrophages, monocytes, etc.) and rapidly proliferates, causing an excessive cascade release of cytokines such as interleukin (IL)-1, IL-6, tumor necrosis factor-alpha (TNF-α), and granulocyte-macrophage colony-stimulating factor (GM-CSF), forming a “cytokine storm”.^415–417^ CRS typically occurs within hours to 14 days after CAR-T cell infusion, with diverse clinical manifestations that can lead to shock, multi-organ dysfunction, and even death, underscoring the importance of early recognition and management of CRS.^418,419^ CRS is less common in non-tumor diseases because the target cell load is much lower than in tumor diseases. Therefore, the application prospects are better compared to tumor diseases, although CRS is not completely absent.^114,177,218^ In clinical trials targeting IIM patients, it has been mentioned that during the first three days, patients showed transient increases in creatine kinase and inflammation-related markers.^218^ Although these markers later returned to normal levels, reaching treatment standards, effective management of CRS is still necessary. Some trials also observed moderate CRS in non-tumor diseases, which exacerbated pre-existing respiratory difficulties.^420^ Therefore, CRS still requires attention in patients with underlying conditions.

L-CRS and LICATS: B-cell non-Hodgkin lymphoma (B-NHL) exhibits distinct pathophysiological features and clinical manifestations compared to other hematologic malignancies such as B-cell acute lymphoblastic leukemia (B-ALL). A notable difference is that B-NHL lesions are typically localized. In B-NHL patients undergoing CAR T-cell therapy, compartmental inflammation can be observed, presenting as redness, swelling, and enlargement around the local lymphoma or lesions. This localized inflammatory response is defined as local CRS (L-CRS).^421^ The activation of monocytes/macrophages and the progression of CRS are likely associated with the in vivo distribution of CAR T cells, and the aggregation of these cells triggered by direct contact activation is a key pathogenic mechanism. Recently, a study comprehensively analyzed existing data on CAR-T therapy-specific adverse events in autoimmune diseases, documenting 54 cases of localized reactions termed Local Immune effector Cell-Associated Toxicity Syndrome (LICATS). LICATS is observed in autoimmune disease patients receiving CD19-targeted CAR T-cell therapy, most likely stemming from the clearance of immune cells from affected organs. It is characterized by self-limiting, organ-specific manifestations that are typically mild in intensity. LICATS reflects differences in toxicity profiles between CAR T-cell therapy for B-cell malignancies and autoimmune diseases. Given the high burden of target cells in lymphoma—which is associated with CRS and ICANS—and the significantly greater tumor cell load compared to the B-cell burden in autoimmune diseases, these CAR-driven inflammatory syndromes are more prevalent and severe in B-cell malignancies than in autoimmune diseases. Consequently, higher-grade CRS and ICANS are uncommon in autoimmune disorders. These self-limiting inflammatory events may arise from the direct effects of CAR T cells, localized cytokine release following target cell killing, or the activation of autoreactive T cells that are reinfused as non-engineered T cells and expand alongside CAR T cells in the patient. Alternatively, they may result from repopulating immune cells after lymphodepletion, including but not limited to the effects of fludarabine on myeloid cells and regulatory T cells. However, given the localized nature of LICATS, its pathogenesis could also involve inflammatory responses related to the clearance of dead B cells and debris in affected tissues. LICATS represents an immune reset-mediated toxicity that is organ-specific, typically emerging days to weeks after CAR T-cell therapy, and most importantly, it is self-limiting and serves as a significant indicator of actual disease recurrence. Unlike CRS, LICATS is likely driven by immune-related clearance processes in tissues previously affected by autoimmune disease prior to CAR T-cell therapy.^422^ The underlying mechanisms of L-CRS and LICATS remain unclear, and further studies with larger sample sizes are needed to advance understanding in this area.

Immune effector cell-associated neurotoxicity syndrome (ICANS): ICANS manifests clinically as toxic encephalopathy, and in severe cases, it can progress to seizures, cerebral edema, motor dysfunction, altered consciousness, and coma.^415,423^ It is a disorder characterized by a pathologic process involving the central nervous system following any immune therapy that results in the activation or engagement of endogenous or infused T cells and/or other immune effector cells. Symptoms or signs can be progressive and may include aphasia, altered level of consciousness, impairment of cognitive skills, motor weakness, seizures, and cerebral edema.^415^ ICANS is commonly observed in tumor diseases due to the target cell load being much lower than in tumor diseases. It is important to note that in non-tumor diseases, particularly neurological disorders, the pathogenesis involves pathogenic cells and antibodies crossing the blood-brain barrier to reach the central nervous system, triggering an inflammatory response and causing cellular dysfunction.^113,302,424^ Therefore, when designing CAR-T therapy, consideration is given to the ability to cross the blood-brain barrier and generate related immune responses in the central nervous system,^304^ which theoretically could lead to ICANS. Although there are currently no clinical trials targeting the nervous system, this remains an important factor to consider.

Differences in peak expansion: compared to the robust expansion observed in hematologic malignancies, CAR-T cell expansion in non-oncologic indications typically exhibits a more moderate peak. This attenuated response can be attributed to several factors: lower antigen burden: in non-oncologic diseases (e.g., systemic lupus erythematosus, systemic sclerosis), target antigens (such as CD19 or BCMA) are expressed at lower levels than on tumor cells, reducing CAR-T cell activation.^176^ Distinct immune microenvironment: Non-oncologic tissues lack the strong co-stimulatory signals (e.g., CD28/CD80 pathway) characteristic of tumor microenvironments, limiting CAR-T cell activation.^425^

Cardiovascular toxicity: Cardiovascular toxicity is not uncommon in patients receiving CAR-T cell therapy. A recent pharmacovigilance study based on the FDA Adverse Event Reporting System reported 2.8% arrhythmias, 2.6% cardiomyopathy, 1.8% cardiogenic shock, 1.7% pleural disease, 0.4% pericardial disease, and 1.6% venous thromboembolism events.^426^ Serious events such as myocardial infarction, atrial arrhythmias, congestive heart failure, and cardiac arrest are generally rare ( < 0.5%). Although the exact incidence of cardiovascular toxicity with CAR-T cell therapy may vary, multiple large centers, trials, and registry studies have reported cardiovascular adverse events.^426–430^ The results of cardiac disease during tumor treatment indicate that troponin measurement can be used as a mean to identify patients at higher risk of cardiac events after CAR-T cell infusion. It may also be attributed to the elderly population, who have higher cardiovascular risk factors and have previously received cancer-related treatments.^431,432^ In the treatment of non-tumor diseases, the use of patients with existing high-risk cardiovascular system factors should also be considered. Currently, some CAR-T cells are also treated with lymphocyte clearance before being used in non-tumor diseases, which may also increase the associated risks.

Targeting non-diseased cell toxicity: similar to the effect seen in tumor diseases where CAR-T cells target non-tumor cells, this refers to CAR-T cells attacking healthy cells that are not pathogenic or non-target cells expressing the same antigen, leading to damage of normal function. This requires more precise CAR design, identification of highly specific targets, and the addition of control systems to CAR-T cells to reduce damage to non-diseased cells. Using tissue-specific isoforms can reduce off-target toxicity in other unrelated tissues.^433^ Although the preservation of pre-existing humoral immunity and specific elimination of pathogenic B cells have been reported for CD19 targeting CAR-T cells,^434,435^ it is crucial to ensure that utilizing CAR-T cells for rheumatic diseases does not result in excessive on-target off-disease toxicity and subsequent damaging of healthy tissue.

Although the CRISPR gene-editing system has shown immense potential in the field of CAR-T immunotherapy, it also introduces additional risks of genetic toxicity.^436–438^ Due to genomic similarity,^439,440^ Cas proteins may induce unintended double-strand breaks (DSBs) at off-target sites, leading to unexpected mutations—commonly referred to as off-target effects. These unintended genetic modifications can result in unpredictable alterations in gene function or regulation.^437^ CRISPR-induced DSBs can cause chromosomal abnormalities, including large deletions, inversions, and translocations. One commonly used approach is TRAC gene knockout; however, studies have reported that it frequently leads to chromosomal aberrations. Editing of the TRAC locus in human primary T cells using CRISPR RNPs has shown that approximately 5–20% of T cells exhibit either large deletions or complete loss of chromosome 14.^441^ Transcriptome sequencing further reveals that these chromosomal deletions lead to abnormal expression of various genes, such as CD70 and MDM2. The Cas9-induced chromosomal loss triggers a p53-associated DNA damage response, activation of apoptotic pathways, and p53-induced cell cycle arrest, ultimately compromising the survival advantage of CAR-T cells.^441^

Additionally, chromosomal translocations pose a serious threat to genomic stability and are recognized as hallmarks of oncogenesis. There have been reports of secondary malignancies following autologous CAR-T cell therapy, raising concerns about the safety of this treatment approach.^442^ Furthermore, the use of viral or plasmid vectors can result in prolonged or permanent nuclease expression, further increasing the risk of genetic toxicity and DNA damage responses.^443,444^

### Effectiveness and sustainability

Autoimmune diseases are characterized by the mediation of multiple autoantibodies. The exact mechanisms of these diseases are not yet fully understood, leading to unknown or inaccurate target. Compared to oncology, the durability and efficacy of CAR-T cell therapy remain a challenge and may extend to its application in autoimmune diseases. According to current research, although CD19 CAR-T cells show significant complete remission rates for relapsed/refractory B-cell and B-cell lymphoma, 40–60% of patients experience disease recurrence.^445,446^ The fundamental reason is that over time, CAR-T cells are prone to exhaustion, leading to reduced anti-tumor effects, cancer persistence, and relapse. The exhaustion issue may reduce the therapeutic effects against pathogenic autoimmune cells, resulting in persistence and relapse.^447^ Furthermore, CAR-T cells target only a selected number of antigens, which could lead to incomplete elimination of the tumor population or allow the disease to escape these antigens, thus contributing to cancer persistence and relapse.^448^ Therefore, it can be imagined that CAR-T cells may not be able to address the heterogeneous nature of the multiple antigens in autoimmune diseases, potentially resulting in disease persistence and relapse years later. Although current clinical trials for non-tumor diseases have shown complete remission, with the longest follow-up being 46 months,^115^ the target B-cell population remains in remission. Reports have observed that 15 ARDS patients received CD19 CAR-T therapy and demonstrated excellent disease remission within months. Studies have reported that 15 ARDS patients treated with CD19 CAR-T therapy demonstrated favorable disease remission over several months. Research on relapse following CAR-T treatment for non-oncological diseases documented a 45-year-old woman with treatment-refractory Jo-1-associated antisynthetase syndrome who achieved disease remission after initial CD19 CAR-T cell therapy, but experienced relapse after 9 months. Upon reinfusion of the same product, the CAR-T cells failed to expand. Despite full-dose lymphodepletion, no effective clinical response was observed. Subsequent combination therapy with the anti-CD38 antibody daratumumab (which showed efficacy but limited durability) and B-cell maturation antigen (BCMA)-targeted CAR-T cells (designed to eliminate plasma cells) was implemented. The BCMA CAR-T cells successfully expanded, cleared plasma cells in lymphoid tissues, reduced autoantibody levels, and re-induced stable drug-free remission. This observation provides insights into achieving sustained remission through CAR-T cell therapy.^449^ Predictably, these issues will need to be addressed.^149^

Functional silence despite long-term persistence has been observed in certain autoimmune diseases (e.g., anti-CD19 CAR-T therapy for systemic lupus erythematosus), where sustained clinical remission persists even when CAR-T cells become undetectable in peripheral blood. This suggests immune reset: transient elimination of pathogenic B cells by CAR-T cells may lead to reconstitution of a non-autoreactive B-cell repertoire, eliminating the need for prolonged CAR-T presence. Tissue-resident localization: CAR-T cells may migrate to and persist in lymphoid organs or inflammatory sites, evading peripheral blood detection while maintaining localized activity.^450^ Notably, unlike in oncology (where CAR-T persistence directly correlates with relapse prevention), therapeutic efficacy in non-malignant diseases often relies on CAR-T-induced immune modulation rather than continuous cytolytic activity. For example, in systemic sclerosis treated with anti-CD19 CAR-T cell, disease improvement continues post B-cell reconstitution, potentially through restoration of regulatory T-cell (Treg) function.^149^ Therefore, this is also the direction that needs to be promoted in the future research on CAR-T cells for non-tumor diseases, such as the construction of CAR-Treg, research on memory CAR-T cells, and how to construct immune reconstitution effects on non-B cell-induced diseases.

### CAR-T cell manufacturing

The manufacturing of CAR-T cells is time-consuming, complex, and expensive due to the complexity of genetic modification, the patient-specific nature of the treatment, the need for strict quality control measures, and the requirements for specialized facilities to produce and handle these cells.^451,452^ When using viral vectors, this complexity is part of the current standard gene transfer method for CAR-T cell production. Specifically, the initial phase of manufacturing the viral vectors for gene transfer typically requires extensive cell culture operations and safety testing, which may take six months to a year to obtain the ready-to-use vectors. Subsequently, there is a risk of contamination from replicating viruses or other unintended viral agents.^453^ After this initial phase, the actual CAR-T cell manufacturing process takes one to two weeks, during which multiple time-sensitive and complex steps are involved.^448^ As a result, the cost of CAR-T cell therapy for each cancer patient is approximately $373,000 to $475,000, with additional costs including extra procedures, patient monitoring, and facility fees.^454,455^ The FDA has approved CAAR-T cancer therapy targeting CD19. However, a major limitation of this new therapy is its high cost. The application fees for related drugs are extremely high, posing a challenge for the widespread use of such medications.^456^

Anti-CD19 CAR-T cells can eliminate various B cells, including non-pathogenic B cells and autoreactive B cells. To more selectively target B cells involved in autoimmune diseases driven by autoantibodies with clearly defined self-antigens, CAAR T cells are designed by expressing pathogenic self-antigens as the extracellular domain of the chimeric immune receptor. This approach is most suitable for diseases where the autoimmune antigen spectrum is limited and the primary pathogenic role of autoantibodies has been clearly defined.^457^ Shortly after the initial report of CAR-T therapy, cluster regularly interspersed short palindromic repeats (CRISPR) editing achieved breakthrough progress. This precise genome editing capability has improved the performance and lifespan of synthetic T cells. CRISPR-edited CAR-T cells have entered clinical trials and are undergoing safety and feasibility evaluations.^413,458^ Recent advances in gene editing technologies, such as base editing or prime editing, have made it possible to generate gene-edited CAR-T cells without introducing double-strand breaks.^459^ Synthetic cell circuits offer significant potential for enhancing CAR-T therapy. These engineering strategies provide opportunities to enhance efficacy, mitigate toxicity, or regulate CAR expression. The latest study showed that CRISPR edited genetic engineering healthy donor-derived CD19-targeted CAR-T cells can solve the problem of immune rejection. These cells were used to treat a patient with refractory immune-mediated necrotizing myopathy and two patients with diffuse cutaneous systemic sclerosis. The infused cells lasted for more than 3 months, and complete B cell depletion was achieved within 2 weeks of treatment. At 6 months of follow-up, we observed that all patients had deep remissions, without cytokine release syndrome or other serious adverse events.^413^ Current CAR-T therapies are limited to targeting surface-bound proteins and glycans. However, recent findings suggest that CAR-T cells can target peptides presented by the major histocompatibility complex. Natural killer cells, macrophages, dendritic cells, B cells, and γδ T cells have shown preclinical potential.^460^ Compared to traditional αβ T cells, such cell engineering efforts may reduce the risk of GvHD and cytokine release syndrome (CRS).

The delivery method of CRISPR components remains a major bottleneck in somatic genome editing.^436^ While traditional viral vector-mediated CRISPR delivery exhibits high efficiency, it is limited by cargo capacity and safety risks, making it difficult to meet the growing demand for improved quality and reduced CAR-T cell production costs. Additionally, compared to ex vivo approaches, in vivo delivery offers advantages such as simplicity, cost-effectiveness, and direct clinical application. However, it still faces significant challenges in transitioning to practical applications.^461^ Nanotechnology and its derivatives have brought innovative strategies to address the existing challenges in the medical field. By designing drug delivery carriers and integrating biological functional molecules, nanotechnology can not only reduce the cost of CAR-T cell preparation but also play a key role in improving their penetration and infiltration in tumors, modulating the immunosuppressive environment of solid tumors, and reducing toxic side effects.^462,463^

### Combination therapy

In cancer treatment, conditioning chemotherapy prior to CAR-T cell therapy is generally considered necessary to ensure the efficacy of the infused T cells through various mechanisms, including the depletion of Treg cells, increased availability of serum cytokines (such as IL-15 and IL-7), and potentially the depletion of myeloid-derived suppressor cells. Several conditioning chemotherapy regimens have been used prior to CAR-T therapy, including cyclophosphamide monotherapy, cyclophosphamide and fludarabine combination therapy, or bendamustine.^464,465^ Compared to cyclophosphamide alone, the cyclophosphamide-fludarabine combination therapy may improve CAR-T cell expansion and clinical outcomes. Therefore, in cancer treatment, the low-dose cyclophosphamide-fludarabine regimen is most commonly used in commercially available CAR-T cell therapies.^149^ The optimal treatment regimen for patients with autoimmune diseases is still unclear, but cyclophosphamide-fludarabine has been successfully used in anti-CD19 CAR-T cell therapy for autoimmune diseases. This combination therapy is generally well-tolerated during the acute phase, with the most common chemotherapy-related adverse effects being mild nausea, fatigue, and expected cytopenia. Compared to cancer indications, the benefit-risk profile for autoimmune diseases differs, so there is a need to explore conditioning regimens with lower toxicity. Fludarabine may deplete Treg cells, which could be harmful in autoimmune diseases.^466,467^

### Cost of CAR-T cells

Currently, CAR-T cell therapies are available only for hematologic diseases. The U.S. Food and Drug Administration (FDA) has approved two CD19-specific CAR-T cell products: Axi-Cel and Tisagenlecleucel (Kymriah, Novartis), priced at $373,000 and $475,000, respectively. Some institutions have conducted analyses on pricing and cost-effectiveness.^37,468^ A quantitative analysis of the cost and benefits of Axi-Cel, assuming a five-year relapse-free survival endpoint for hematologic malignancies, compared the one-year mortality rate of patients in remission with that of the general population of the same age group. The analysis projected that such patients could gain an additional 28 years of life expectancy—a result that is clearly overly idealized. This highlights the current data limitations, making it difficult to achieve sample-matched or comparative cost-benefit analyses in the short term.^469^ As a result, patients must currently decide whether to undergo CAR-T therapy based on their individual needs and financial capacity. However, despite the high costs, these therapies offer hope and significant treatment potential, warranting further development and broader accessibility.

## Prospective of CAR-T cell therapy in non-oncological diseases

### Emerging technologies

The development of combination CAR-T strategies stems from a recognition of the limitations inherent in single-target therapies. This is particularly evident in B-cell lymphomas, where monotherapy targeting CD19 often leads to relapse due to the loss of the target antigen (antigen escape)^470^; however, in multiple myeloma (MM) patients treated with CAR-T therapy, BCMA loss was rarely observed or only suspected.^471^ This raises the assumption of whether multi-target strategies can be combined for treatment. Multi-target CAR-T strategies, such as tandem/dual, bispecific, or co-administration/co-transduction CARs, as well as sequential approaches targeting CD19/CD22 or CD19/CD20, have been investigated in clinical studies of B-ALL as alternative methods to avoid relapse.^58,472–476^

The severity of SLE/LN is associated with an increased expression of the BCMA surface antigen on long-lived plasma cells, making it a promising therapeutic target. Unlike the CD19^+^ cell population, CD19^−^ long-lived plasma cells reside in the bone marrow. Anti-double-stranded DNA (dsDNA) antibodies are produced by both CD19^+^ and CD19^−^ plasma cells. A dual CAR approach, consisting of two independently functioning CARs that target CD19 on B cells and BCMA on long-lived plasma cells, holds the promise of eliminating the cell types that produce disease-defining autoantibodies. This strategy could potentially address the root cause of the disease and lead to a reset of both B cell and humoral immunity.^115^

Universal CAR-T cells to reduce costs: The use of allogeneic CAR-T cells derived from donors offers several potential advantages, such as the availability of cryopreserved batches for immediate patient treatment, standardization of CAR-T cell products, the possibility of simultaneous modifications to multiple cells, and reduced costs or streamlined industrial processes for CAR-T cell combinations targeting different antigens. However, allogeneic CAR-T cells may cause life-threatening graft-versus-host disease and could be rapidly eliminated by the host immune system. Thus, the development of next-generation allogeneic CAR-T cells is an active area of research.^172^

Autologous CAR-T cell therapy requires a personalized manufacturing process for each patient following leukapheresis. Although this approach has demonstrated excellent clinical data to date, it also has significant drawbacks, such as high costs, manufacturing failures in some patients, and a manufacturing process that takes approximately 3 weeks.^477^ This delay can affect treatment availability or result in treatment failure due to dysfunctional autologous T cells. Consequently, the high treatment costs place a substantial burden on both patients and the healthcare system.^478^

### Immune remodeling effects

A core feature of the immune system is its ability to generate protective immunity upon re-exposure to the same antigen. This immunological memory prevents reinfection by the same pathogen and serves as the foundation for vaccine efficacy and the control of adaptive abnormalities, including cancer. However, this immune memory also has inherent limitations. When central and peripheral tolerance mechanisms fail to prevent immune responses against self-antigens or harmless antigens, immunological memory plays a pivotal role in sustaining inflammation. This persistence becomes a key obstacle to achieving long-term remission or cure in allergies and autoimmune diseases. Immune resetting in autoimmune therapy refers to the elimination of this immune memory to “remodeling” the immune system, restoring its normal function and halting attacks on self-tissues.^479^ Targeting memory B cells offers a promising pathway for immune resetting, potentially enabling the cure of autoimmune diseases. This approach has already received preliminary clinical validation. For example, the CD20 monoclonal antibody rituximab has demonstrated efficacy in treating rheumatoid arthritis and improving systemic lupus erythematosus (SLE), providing early evidence supporting B-cell depletion as a therapeutic strategy for autoimmune diseases.^480^ To ultimately cure autoimmune diseases, deep and sustained B-cell depletion is theoretically required. Emerging therapies, including cell-based treatments and bispecific antibodies, may leverage the persistence of T cells to achieve this goal. In 2022, CAR-T therapy clinically validated this concept. A German investigator-initiated trial (IIT) demonstrated that five refractory SLE patients treated with CD19 CAR-T cells achieved rapid and sustained B-cell depletion alongside normalized immune parameters. Notably, these patients maintained long-term treatment-free remission.^114^

Patients with relapsed or refractory hematologic cancers generally have poor prognoses. Chimeric antigen receptor (CAR) T-cell therapy serves as a bridge to allogeneic hematopoietic stem cell transplantation (HSCT) and has the potential to achieve long-term tumor eradication. Standalone allogeneic HSCT often leads to severe graft-versus-host disease (GvHD), whereas CAR-T therapy offers a novel approach by clearing both tumor cells and certain immune cell populations in hematologic malignancies.^481^ The use of universal CD7 CAR-T cells has been associated with manageable safety and encouraging antitumor activity in early reports,^482,483^ supporting their role in reshaping the immune environment before HSCT. When used as consolidation therapy, HSCT can maintain long-term disease remission and reduce the incidence of GvHD.^484–486^ In a study evaluating this sequential approach, all 10 patients successfully controlled cytokine release syndrome (CRS), with no cases of immune effector cell-associated neurotoxicity syndrome (ICANS). Three patients developed grade 2 acute GvHD, but no cases of chronic GvHD were observed.^168^ With the successful application of allogeneic CD19 CAR-T cells in autoimmune diseases,^413^ future strategies integrating universal CAR-T therapy with HSCT to sustain immune modulation may offer significant benefits for autoimmune and other chronic diseases.

Conventional antigen-specific Treg cells may inhibit effector T cells specific to other antigens through the production of soluble mediators or direct cell-to-cell interactions—this phenomenon is known as bystander suppression. Studies have shown that in T1D, Treg cells expressing autoreactive TCRs can induce bystander suppression, as the local activation of homologous antigens inhibits the proliferation of effector T cells specific to another T1D autoantigen. Therefore, the suppressive state may be self-sustaining when transferred from engineered Treg cells to endogenous T cells, suggesting that engineered Treg cells may not necessarily require long-term persistence.^487^

### Biomarker-driven therapy

Biomarkers are measurable and quantifiable biological molecules, including proteins, DNA, RNA, or biochemical compounds, that serve as indicators of specific biological states or conditions. In the context of personalized cancer therapy, biomarkers help determine patient prognosis and predict which patients will respond to a chosen therapy or experience adverse side effects from specific treatments.^488^

For non-tumor diseases, biomarkers assist in understanding disease mechanisms, helping to identify the target cell interactions of CAR-T therapy. For example, senescence-associated secretory phenotype (SASP) is strictly regulated by cellular senescence and plays a crucial role in development and tissue repair. SASP exerts its effects through the secretion of chemokines, which recruit inflammatory cells such as macrophages to remove senescent cells and restore normal tissue function. SASP consists of multiple cellular factors, including cytokines, chemokines, and matrix metalloproteinases (MMPs).^370,489^ The senescence mechanism manifests at the organ or tissue level. For instance, in the lungs, senescent lung cells secrete proteins that induce neighboring cells to undergo senescence or activate fibroblasts, leading to pulmonary fibrosis, which compromises lung function. Similarly, astrocytes can induce neuronal cell death, resulting in neurological dysfunction.^36^ Building on research into senescence mechanisms, scientists have designed uPAR CAR-T cells, which can recognize the secretory phenotype of senescent cells and eliminate them, thereby maintaining normal organ and tissue function.^36,368^ Since CAR-T cell therapy itself is a highly individualized treatment—requiring precise gene editing to recognize specific antigen structures—biomarkers remain a hot research topic in this field.^490^

The success of CAR-T cell therapy hinges on finding and developing highly specific, strongly stimulatory target antigens, such as the currently researched hot targets like mesothelin (MSLN), epidermal growth factor receptor (EGFR), prostate-specific membrane antigen (PSMA), and glypican 3 (GPC3).^460^ Additionally, the development of multi-target antigen CARs has achieved promising therapeutic effects in addressing challenges such as antigen loss density decline leading to resistance recurrence and solid tumor antigen heterogeneity. Cell therapies targeting CD20, CD22, and CD123 combined with CD19 have shown low toxicity and high efficiency in clinical trials.^57^ Another direction for multi-target development is to continue optimizing CAR structures, addressing the challenges faced by dual-specific CAR-T cell therapy, poor efficacy in solid tumors, resistance recurrence, and toxic side effects. The bi-specific T-cell engager (BiTE) has promising development prospects. BiTEs contain two scFvs, one recognizing the T cell surface protein CD3 and the other recognizing tumor cell surface antigens, usually tumor-associated antigens (TAA), thereby activating CAR-T cells and endogenous T cells to kill tumor cells.^491^

## Conclusion

CAR-T therapy holds transformative potential beyond oncology, offering promising applications in autoimmune diseases and other chronic conditions. However, realizing its full potential requires collaborative research efforts to address critical challenges, including safety concerns, high treatment costs, and the mechanistic complexities of immune modulation. By advancing our understanding of CAR-T’s broader therapeutic impact, we can refine its applications and enhance accessibility for a wider range of patients.

With advancements in molecular fields and the integration of medical engineering, there will be better prospects for the design, preparation, and application of CAR-T cells.
